# Conformational Coupling between Receptor and Kinase Binding Sites through a Conserved Salt Bridge in a Signaling Complex Scaffold Protein

**DOI:** 10.1371/journal.pcbi.1003337

**Published:** 2013-11-14

**Authors:** Davi R. Ortega, Guoya Mo, Kwangwoon Lee, Hongjun Zhou, Jerome Baudry, Frederick W. Dahlquist, Igor B. Zhulin

**Affiliations:** 1 Joint Institute for Computational Sciences, University of Tennessee - Oak Ridge National Laboratory, Oak Ridge, Tennessee, United States of America; 2 Department of Physics, University of Tennessee, Knoxville, Tennessee, United States of America; 3 Department of Chemistry and Biochemistry, University of California, Santa Barbara, California, United States of America; 4 Department of Biochemistry and Cell and Molecular Biology, University of Tennessee, Knoxville, Tennessee, United States of America; 5 Center for Molecular Biophysics, University of Tennessee - Oak Ridge National Laboratory, Oak Ridge, Tennessee, United States of America; 6 Department of Microbiology, University of Tennessee, Knoxville, Tennessee, United States of America; University of Illinois at Urbana-Champaign, United States of America

## Abstract

Bacterial chemotaxis is one of the best studied signal transduction pathways. CheW is a scaffold protein that mediates the association of the chemoreceptors and the CheA kinase in a ternary signaling complex. The effects of replacing conserved Arg62 of CheW with other residues suggested that the scaffold protein plays a more complex role than simply binding its partner proteins. Although R62A CheW had essentially the same affinity for chemoreceptors and CheA, cells expressing the mutant protein are impaired in chemotaxis. Using a combination of molecular dynamics simulations (MD), NMR spectroscopy, and circular dichroism (CD), we addressed the role of Arg62. Here we show that Arg62 forms a salt bridge with another highly conserved residue, Glu38. Although this interaction is unimportant for overall protein stability, it is essential to maintain the correct alignment of the chemoreceptor and kinase binding sites of CheW. Computational and experimental data suggest that the role of the salt bridge in maintaining the alignment of the two partner binding sites is fundamental to the function of the signaling complex but not to its assembly. We conclude that a key feature of CheW is to maintain the specific geometry between the two interaction sites required for its function as a scaffold.

## Author Summary

Signal transduction is a universal biological process and a common target of drug design. The chemotaxis machinery in *Escherichia coli* is a model signal transduction system, and the CheW protein is one of its core components. CheW is thought to work as a scaffold protein that mediates the formation of the signaling complex with the CheA histidine kinase and the chemoreceptors. A mutation targeting a highly conserved residue, Arg62, impairs chemotaxis while maintaining normal binding affinity for both partners, suggesting that CheW might play a more complex role than previously proposed. Using a series of molecular dynamics simulations, we found that the residue Arg62 can form a stable salt bridge with another highly conserved residue, Glu38. We determined that this bridge does not contribute to the overall stability of the protein. However, the bridge stabilizes the local backbone structure of CheW and stabilizes the relative position of the binding sites for the chemoreceptor and kinase. The geometry of these interactions appears to be vital for the function of the signaling complex. We validated and complemented our computational findings using NMR spectroscopy and circular dichroism analysis.

## Introduction

The *Escherichia coli* chemotaxis pathway employs dedicated chemoreceptors that are anchored in the membrane and detect signals from both outside and inside the cell [1]. Chemoreceptors relay this information to the CheA histidine kinase, which then communicates the information to its cognate response regulator CheY. In a phosphorylated form, the CheY protein binds to flagellar motors to cause a change in the direction of its rotation, thus converting the initial signal detected by chemoreceptors into a behavioral response – a change in the swimming direction. This pathway also employs the receptor-modifying enzymes CheB and CheR as well as the CheZ phosphatase, which acts on CheY [2].

The key features of this remarkable system include high sensitivity, wide dynamic range, signal integration, memory, and precise adaptation [3]–[7], all of which are consequences of a highly ordered arrangement of chemoreceptor and kinase proteins at the cell pole [4], [8], [9]. The geometry of a hexagonal array with a lattice spacing of 12 nm is conserved over long evolutionary distances [9], indicating the importance of precise interactions among members of the complex. In addition to the chemoreceptors and the CheA kinase, this complex also contains the CheW protein, which is interchangeably referred to as a docking, scaffold, coupling, or adaptor protein [10]–[12].

The crystal structure of CheW [10], [13], [14] reveals a fold composed of two five-stranded β-barrel subdomains connected by a hydrophobic core. Within the chemotaxis signaling complex, the CheW fold is present not only as a stand-alone adaptor but also as a homologous domain within the CheA kinase [15]. Furthermore, the two subdomains of CheW are topologically similar to the SH3 domain [15], which is widely distributed among scaffold proteins in eukaryotic signal transduction systems [16]. Thus, elucidating the structure/function relationships of CheW will have a broader impact in understanding the role of scaffold proteins in signal transduction system in all organisms.

CheW is required for proper activation of the kinase by the chemoreceptor [17] and is essential for the formation of the chemotaxis complex [18]. Overexpression of CheW disrupts formation of chemoreceptor trimers by blocking trimer contacts [12], [19], [20], thereby impairing chemotaxis [21]. The binding sites for CheA and the chemoreceptor on CheW have been mapped using various experimental approaches [12], [19], [22]–[24]. The overall results were consistent with CheW being a scaffold protein. However, the replacement of Arg62 (throughout the text, numbers are for *E. coli* CheW) with His, which moderately affected in vitro binding affinity of CheW for both its binding partners, completely abolishes chemotaxis. This finding indicates that CheW plays a role in addition to holding CheA and the chemoreceptors together [24].

Our view on the role of scaffold proteins in signal transduction is rapidly changing. They can no longer be viewed as nothing more than molecular “glue”. It is clear that their dynamics must play a central role in their communication with partner proteins in signaling proteins [25]. Although X-ray and NMR structures are excellent starting points, they do not describe the dynamics properties of proteins. These properties can be studied only by tracking the time-dependent positions of all atoms in the system through molecular dynamics simulations, a methodology that has improved dramatically in recent years [26].

In this study, we sought to gain a deeper insight into the structure/function relationship of CheW by using a combination of sequence analysis, NMR spectroscopy, circular dichroism (CD), and molecular dynamics (MD). This approach revealed the existence of an evolutionarily conserved salt bridge on the surface of CheW that is responsible for maintaining the stability of a specific geometry within the signaling complex that is essential for its function.

## Materials and Methods

### NMR spectroscopy

NMR data were collected at 30°C with a Varian Inova 600 MHz spectrometer equipped with a four-channel (^1^H, ^13^C, ^15^N, and ^2^H) cryoprobe and Z-axis pulsed field gradients. NMR data were analyzed with the nmrPipe package and ANSIG3.3 [27], [28]. The wild-type CheW backbone chemical-shift assignments were obtained from previous publication (BMRB accession No. 15322) [13]. Of the 154 published assignments, 123 were transferred to our wild-type CheW ^15^N-HSQC spectrum. The remaining assignments (20%) were not transferred because of the overlap or the weak intensity of these resonances under these experimental conditions. All NMR samples were analyzed in 30 mM Tris-HCl (pH 7.3), 30 mM NaCl, 0.2% sodium azide in 90% H_2_O and 10% D_2_O. The concentration of the NMR sample was 1 mM for the WT CheW and 1.5 mM for the R62A mutant.

The longitudinal relaxation time T_1_ (or inverse rates R_1_), transverse relaxation time (or inverse rates R_2_), and the ^1^H-^15^N NOE factor of backbone amide ^15^N nuclei were measured using inverse-detected two-dimensional (2D) experiments [29]–[33]. Measured delay times for R_1_ relaxation rate were 11, 55, 110, 220, 330, 440, 660, 880, and 1210 ms. Measured delay times for R_2_ relaxation rates were 16.5, 33, 49.5, 66, 82.6, 99.1, 115.6, 132.1, and 148.6 ms. A recycle delay of 1.5 s was used for both R_1_ and R_2_ measurements. R_1_ and R_2_ were extracted by fitting the peak intensities with a single exponential-decay function. The ^1^H-^15^N NOE factor was taken as the ratio of the peak intensities with and without proton saturation during 3 s of the 8 s recycle delay period [33], [34].

Further analysis of the dynamics data was performed by using the MODELFREE program [30], [32], [35], [36] to provide information on the internal and overall motions. The ^15^N R_1_, R_2_ and ^1^H-^15^N NOE values were fitted to a single isotropic rotational diffusion model described by the overall correlation time τ_m_. The model contains a contribution from fast internal motions described by the order parameter S^2^ and the correlation time τ_e_ and from additional exchange broadening (R_ex_) on the time scale of µs to ms. During the calculation, τ_m_ was fixed at 11.0 ns for wild-type and 11.6 ns for the mutant, and internal motional parameters were optimized [29]–[32], [35]–[38].

For more accurate characterization of the chemical exchange contribution (R_ex_) to the transverse relaxation rate constant, a series of modified Carr-Purcell-Meiboom-Gill (CPMG) relaxation-dispersion experiments were performed [39]–[41]. The total CPMG period was kept constant at 80.0 ms while the delay τ_cp_ was varied for a total of 9 values ranging from 1.0 ms to 20.0 ms. The ΔR_ex_ term, with a base value at the fastest spin-echo rate or the shortest τ_cp_ = 1 ms, can be extracted by the following equation:
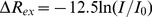
(1)where I is the peak intensity at τ_cp_ and I_0_ is the peak intensity with τ_cp_ = 1 ms. The value of R_ex_ is determined by the difference in chemical shift between two exchange sites (Φ_ex_) and the reduced lifetime of the exchange sites (τ_ex_):
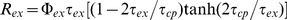
(2)in which 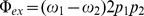; and p_i_ and ω_i_ are the population and Larmor frequency for the nuclear spin at site i, respectively, and τ_ex_ is the reduced lifetime of the exchanging sites [38].

### Circular Dichroism

CD spectra were collected with an Aviv CD Spectrometer Model 202. Wild-type and the R62A mutant variant of CheW were diluted to 7 µM in a 1 cm path-length quartz cuvette. Each sample was then titrated with 11.0 ml of the 7 µM protein and 9.5 M Urea (Amresco, Ultra Pure Grade). The urea-induced denaturation experiments were controlled by a Microlab 500 series dual syringe auto-titrator, and the 220 nm CD signal for each data point was collected at 25°C. Four measurements were collected for the wild-type and two for the R62A variant (Table S1). The data were averaged and normalized. Assuming that CheW wild-type and R62A mutant undergo a two-state unfolding mechanism, the fraction unfolded curve vs. [urea] for each variant was fitted to a six parameters equation [42]:
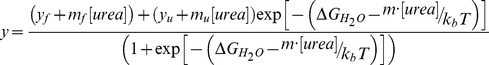
(3)where y is the CD signal, y_f_ and y_u_ are intercepts, m_f_ and m_u_ are the slopes of the pre and post-transition baselines, m is a measure of dependence of ΔG on urea concentration, and 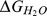 is an estimate of the conformational stability of the protein in 0 M of urea. We used non-linear least square fit to calculate each of the parameters, Table 1.

**Table 1:** Urea denaturation curve analysis for wild-type and R62A mutant.

CheW variant	m_u_	y_u_	m_f_	y_f_	m (kcal/(mol•M))	ΔG_H2O_ (kcal/mol)
**WT**	0.035±0.002	0.66±0.02	0.0346±0.0008	0.016±0.002	1.24±0.03	7.4±0.2
**R62A**	0.035±0.004	0.64±0.03	0.054±0.001	−0.003±0.003	1.32±0.07	7.5±0.4

### Bioinformatics

We collected 3738 CheW protein sequences available from draft and complete genomes, using the August 2012 release of the MIST database [43]. Using HMM provided by the authors [44] and HMMER 2.3.2 [45], we assigned the CheW sequences to classes [44]. We selected only sequences from flagellar systems (2553 sequences), and to avoid contamination by proteins with unassigned or unknown domains in addition to the CheW domain, we used a length filter. Sequences shorter than the Pfam [46] model for CheW (PF01584) or 100 amino acids larger than the model were discarded. Only 368 sequences were discarded in this process. The 2185 CheW sequences selected were separated according to their chemotaxis classes in individual files. Each file was subjected to multiple sequence alignment using algorithm L-INS-I from the package MAFFT [47]. To avoid redundancy, sequences with more than 98% identity were removed from the dataset. The final dataset contained 1429 sequences. Fig. S1 shows the distribution of these sequences in chemotaxis classes. The sets for classes F1 and F7 were used to calculate the identities presented in Table S2.

### Structures and simulation system

The atomic coordinates of *E. coli* CheW were obtained from the NMR structure deposited on PDB (PDB code: 2HO9) [13]. There are 20 frames in the PDB file, and the frame with the lowest alpha carbon RMSD relative to the average of all frames was selected. Standard protocols for solvation and neutralization were used to build the 64×91×70 Å simulation cell with a total of 36193 atoms. After 1000 steps of energy minimization, the frame at 40 ns of equilibration at 298K and NPT ensemble was selected as the starting point for production simulations of the wild-type protein and to build the *in silico* mutants R62A and E38A. To ensure that all three simulation systems (wild-type, mutant R62A, and E38A) were similar, only 200 steps of energy minimization were applied to each of the two simulations cells with mutant proteins.

### Molecular dynamics protocols

All simulations were performed with NAMD2 [48] using CHARMM22 [49] force fields for proteins and the TIP3P model for water [50] in the NPT ensemble. Temperature and pressure were held constant at 298 K and 1 atm using a Nose-Hoover Langevin piston [51] with a period of 100 fs and a decay time of 50 fs. The integration time-stepping was set to 2 fs under a multiple time stepping scheme [52], with bonded and non-bonded interactions calculated at every step, and long range electrostatics interactions calculated at every other step. For the description of the long range forces, van der Waals forces had a cutoff of 12 Å, and the switching function started at 10 Å to ensure smoothness. Electrostatic interactions were calculated using particle mesh Ewald (PME) with a grid-point density of over 1/Å. For the wild-type and both mutant proteins, ten 90 ns-long, independent simulations were produced. In each simulation, atom velocities were reinitialized, guaranteeing independence between runs. The same simulation settings described in the equilibration section were used. The computation was performed using 512 nodes in the Newton Cluster at The University of Tennessee-Knoxville, with a performance of ∼33 ns/day.

### Calculation of the frame-average RMSD per residue

To calculate the frame-average RMSD per residue, we executed the following procedure: (1) from each of the ten simulations with the wild-type structure, the frames in which Arg62 and Glu38 formed a salt-bridge in geometry A were selected. (2) For each one of the ten sets of frames, the RMSD per residue was calculated against the initial frame, which is common to all simulations. (3) The RMSDs per residue were independently averaged over the number of frames in each set. The RMSDs were calculated using the VMD tcl command “rmsd,” and all atoms were taken into consideration.

The same procedure was executed for the simulations with the mutant R62A structure. However, to produce the ten sets of frames, the same number of frames selected from the wild-type simulations (64%) was randomly selected from each of the ten independent simulations. Statistical significance was calculated using two-tailed t-tests for each residue independently.

#### Order parameter calculations

We calculated the order parameter defined by Lipari and Szabo [35]. We use a discrete version of equation 3 in [53]:
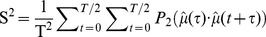
(4)where t and τ scans over the sequence of frames, 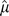 is the unit vector pointing along the backbone N-H bond, and T is the total number of frames. P_2_(x) = ((3x^2^)/2-1/2) is the second Legendre polynomial.

#### General protocol for frame alignments

CheW contains several loops. In our simulation, these loops were very flexible and alignment of the frames was rather poor, which dramatically affected the results of the order parameter calculations. It was therefore important to align the frames using only the most stable regions of the molecule. The residues with the lowest RMSF values per residue calculated from the production part of the initial 280 ns simulation were selected for the alignment. The cutoff was determined by the 75th percentile of the distribution of the calculated RMSF for each residue. As a result, only residues with less than 4.87 Å RMSF were used to align the frames for order parameter calculations: 15 to 43, 46, 47, 53 to 61, 64 to 81, 85 to 119, 126 to 136, 140 to 156.

## Results

### Two highly conserved residues in CheW form a short-range salt bridge

Protein residues in proteins that are conserved over long evolutionary distance usually play the most critical roles in their structure. The signal transduction pathway for chemotaxis originated early in the evolution of bacteria and diversified into many distinct classes, in which the repertoire of interacting proteins can be quite different [44]. For example, in the F1 (F stands for systems that control flagellar motors and a number represents a clade on the chemotaxis phylogenetic tree, see [44] for details) class exemplified by *Bacillus subtilis* and *Thermotoga maritima*, the CheW protein interacts with chemoreceptors that are structurally different from those in the F7 class exemplified by *Escherichia coli* [54]. Furthermore, within a genomic dataset, protein sequences in each class are unequal in numbers and in phylogenetic relatedness, which further complicates analysis. In order to identify residues that are critical to the function of the CheW protein, we assigned the CheW sequences collected from the MIST database [43] to chemotaxis classes and found that F1 and F7, the most abundant classes, are comparable in size (Fig. S1). Therefore, we performed detailed sequence analysis only on the CheW-F1 and CheW-F7 subsets.

**Figure 1:**
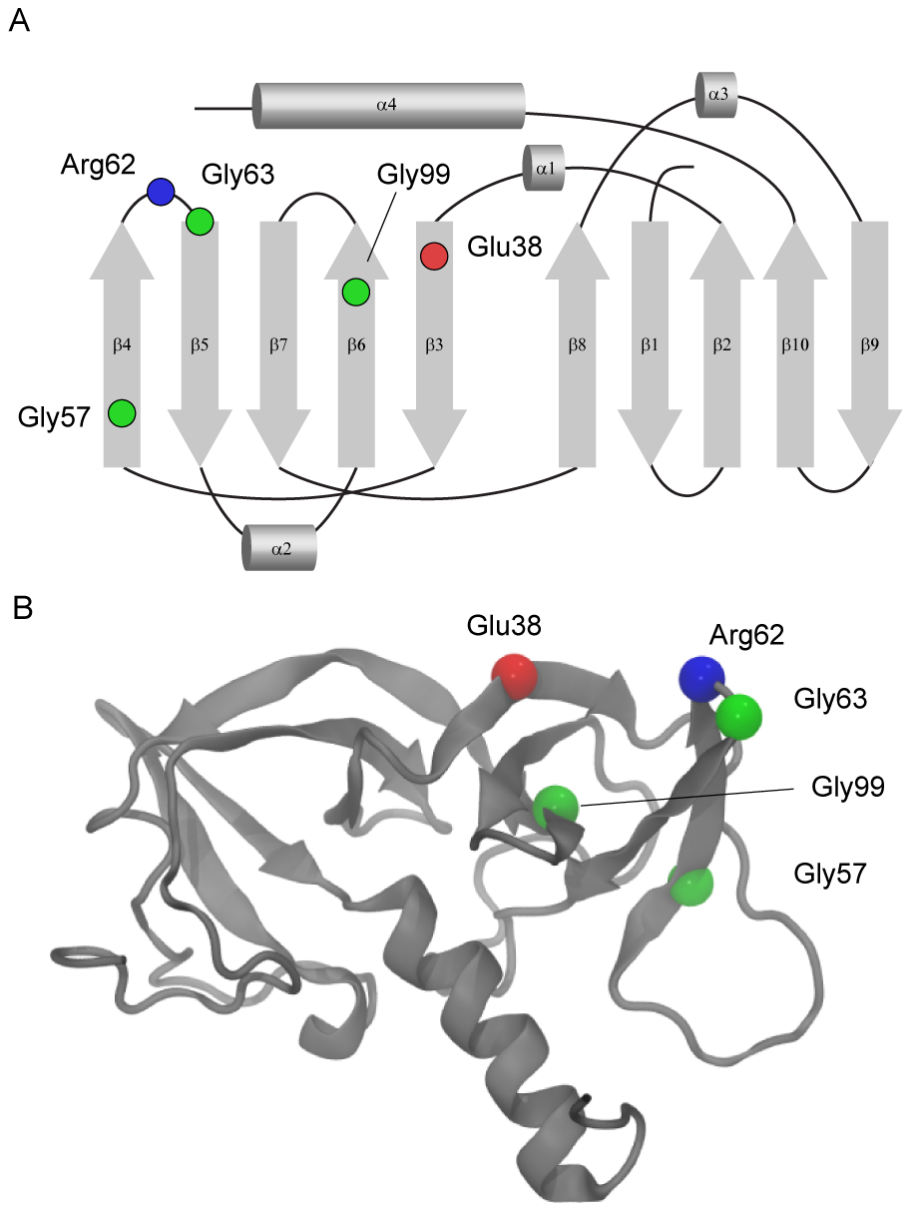
Highly conserved residues in CheW. Mapping of conserved residues on a topology diagram (A) and the three-dimensional structure (B) of *E. coli* CheW.

Earlier analysis of CheW sequences indicated that it is a relatively poorly conserved protein [55]. Thus, it was not surprising to discover that, among the five most conserved positions in each class, only two Gly residues are absolutely conserved in both classes (Table S2). Conservation of a Gly residue usually indicates that it performs unique structural role, either by allowing sharp turns and bends or its location in a spatially constrained environment [56], [57]. Indeed, Gly63 is located at a critical turn on the CheW tertiary structure, and Gly57 is present in a beta sheet bend (Fig. 1). An unexpected finding, however, was a nearly absolute conservation of two charged residues (Arg62 and Glu38 in the *E. coli* protein) in the F7 class (Table S2). We therefore focused our investigation on the properties of CheW-F7, which includes the *E. coli* CheW protein.

Arg62 and Glu38 are in close proximity in the tertiary structure (Fig. 1B). Both Arg62 and Glu38 (along with some other residues) have been implicated as functionally important in previous experimental studies with the *E. coli* protein. Mutations targeting Glu38 reduce the binding constant between CheW and the Tar chemoreceptor, making it a likely candidate for being located in the receptor-binding site [23]. Substitutions in residues in close proximity to Arg62 decrease the binding affinity between CheW and CheA; however, substitutions at Arg62 itself do not appreciably affect binding affinities for either CheW or CheA although they impair chemotaxis [23]. Thus, defining the role of this conserved residue remains a challenge, despite the fact that it has been approached by different experimental techniques [12], [22], [23]. Their physical proximity and their opposite charges suggest that Arg62 and Glu38 residues interact. Furthermore, the highest level of evolutionary conservation of both residues suggests this interaction is critical to protein function.

**Figure 2:**
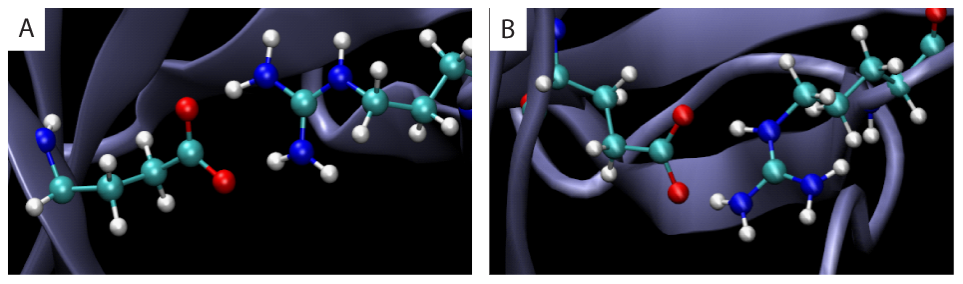
Two conformations of the salt-bridge formed between Arg62 and Glu38 in CheW. In simulations with the wild-type structure, 64% of frames have the residues Arg62 and Glu38 in salt-bridge formation in geometry A (A), and 20% in geometry B (B).

A salt bridge between Arg and Glu can be inferred from an *in-silico* model if the pair of residues meet the following criteria: i) the centroids of the side-chain charged groups are within 4.0 Å of each other; and ii) at least one pair of carbonyl oxygen and side-chain nitrogen atoms are within 4.0 Å of each other. When the ion pair only meets the latter criterion, it is inferred to be forming a N-O bridge [58]. By these criteria, only 4 out of 20 in the ensemble of NMR models resolved for CheW from *E. coli* [13] identify a salt bridge between Arg62 and Glu38. To explore these static models further, we performed ten independent MD simulations of 90 ns each; with a total of 450 thousand frames after an equilibration period of 30 ns (see Materials and Methods). In our simulations, 84% of the frames met both criteria, and 11% met only the latter. In only 5% of the frames were neither of these criteria met. The temporal evolution of the distance between centroids of the side-chain charged groups is shown in Fig. S2. In subsequent analysis, we found two distinct geometries for the salt bridge: (A) atoms NH1 and NH2 are within 4.0 Å of two distinct oxygen atoms in the Glu side-chain, and (B) both atoms NE and NH2 are within 4.0 Å from a different oxygen atom in the Glu side-chain (Fig. 2). In 64% of all frames the residues were in geometry A, and in 20% they were in geometry B.

### Interaction between Glu38 and Arg62 affects protein dynamics

**Figure 3:**
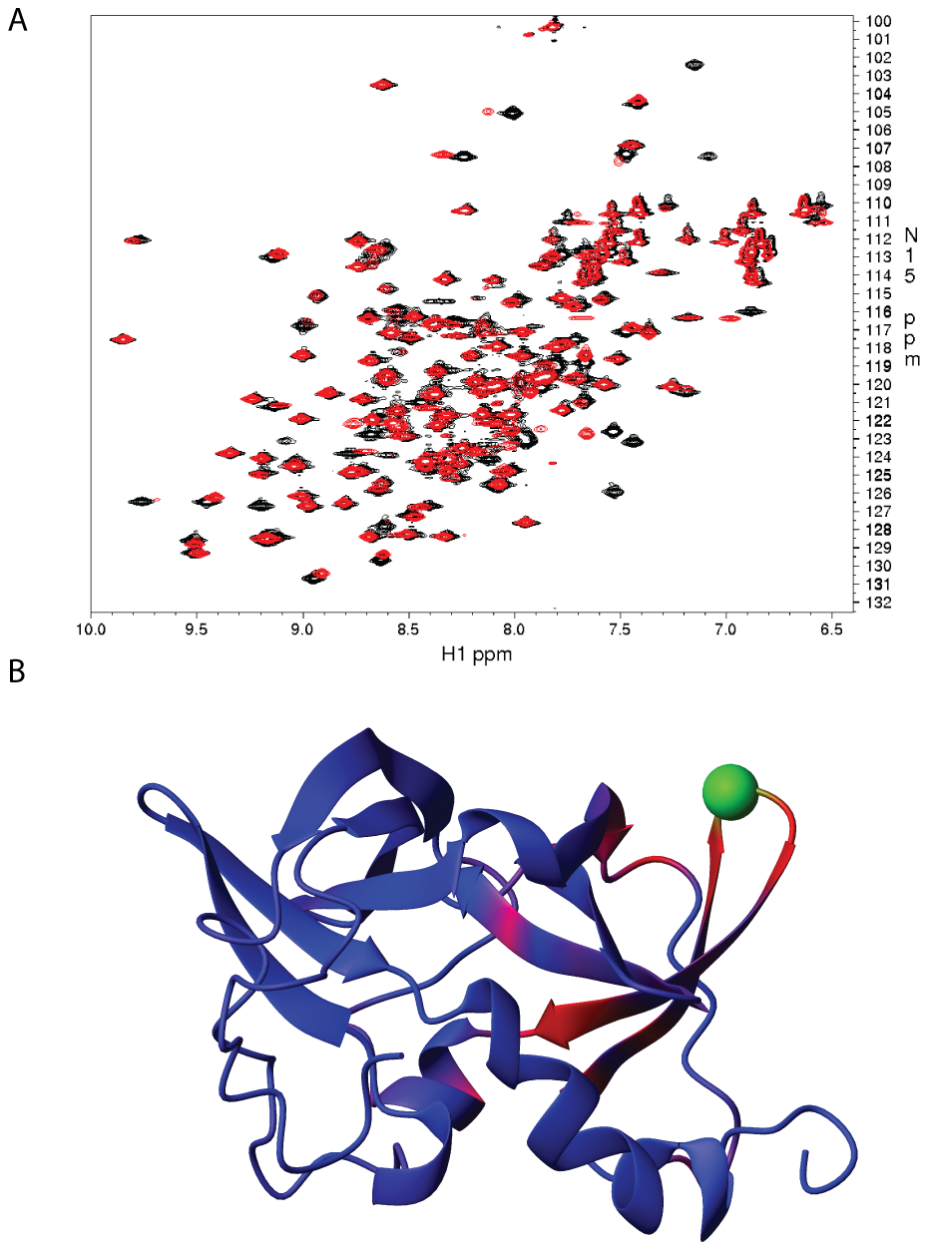
Effects of the mutation R62A on the CheW structure. A) Superposition of 1H-15N HSQC spectra of Wild-type CheW (black) and the mutant CheW R62A (red). B) The chemical shift perturbation between wild-type and R62A CheW color-mapped onto the CheW structure (PDB code 2HO9). The red color indicates larger chemical shift difference and blue color showed smaller differences. The mutation site R62 is shown in green.

To confirm the existence of the salt bridge experimentally, we first attempted to measure the pKa for the wild-type CheW and for a mutant targeting Glu38 using pH titration. Unfortunately, wild-type CheW precipitated at pH less than pH 6.0, which prohibited the use of this method, which requires a larger excursion of pH titration for Glu in proteins [59]. However, because “self-interactions” between residues in a protein molecule are known to contribute to protein dynamics, we attempted to examine the role of Glu38 and Arg62 residues by using NMR. First, we compared the ^15^N-HSQC spectrum of E38A and R62A mutants to that of the wild-type protein (Table S3). The results showed that the E38A mutation caused a global structural perturbation, suggesting that it introduces severe structural changes (Fig. S3). Consequently, we did not pursue further studies with this mutant. On the other hand, the R62A mutation caused only local structural perturbations (Fig. 3) while disrupting the interaction with Glu38. Residues of the R62A mutant that showed significant chemical shift changes are mainly located in β4–β5, the C-terminus of the β-strand containing Glu38 (β3), and residues in close proximity to these limited regions (Table S3).

**Figure 4:**
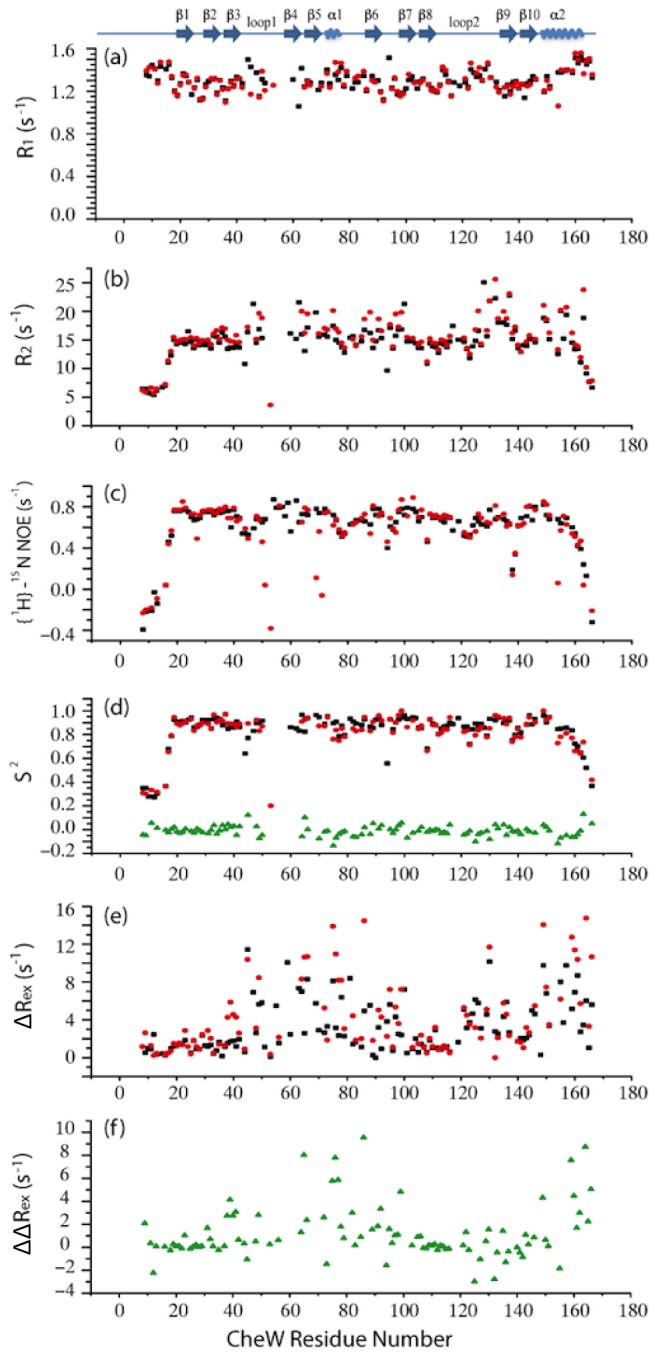
Effects of the mutation R62A on the global and local backbone dynamics of CheW. Backbone amide ^15^N relaxation parameters for CheW vs. residue number are shown. The black squares represent wild-type CheW, the red circles represent R62A mutant, and the green triangles represent the difference between these two constructs. Approximate location of secondary structural elements is shown at the top: (a) the longitudinal relaxation rate R_1_; (b) the transverse relaxation rate R_2_; (c) ^1^H-^15^N NOE; (d) the extracted order parameter S^2^ (e) the differences phenomenological transverse relaxation rate constant ΔR_ex_ = R_ex_(20 ms)−R_ex_(1 ms); (f) the differences between the ΔR_ex_ in (e) ΔR_ex_(R62A)−ΔR_ex_(WT).

To investigate the significance of the interaction between Glu38 and Arg62 in more depth, we measured the relaxation parameters of the backbone ^15^N nuclei in both the wild-type and the R62A CheW proteins. The average longitudinal relaxation rate R_1_ was 1.299 s^−1^ for wild-type and 1.295 s^−1^ for the mutant (Fig. 4a). The longitudinal relaxation is caused by fluctuations at the NMR transition frequencies and reflects the NMR excited state lifetime, which is not altered by the substitution in the position Arg62. The average transverse relaxation rate R_2_ was 14.62 s^−1^ for wild-type and 15.38 s^−1^ for R62A mutant (Fig. 4b). The transverse relaxation reflects any events that cause dephasing of spins in the xy plane, such as rotational diffusion or chemical exchange. Although the absolute values are characteristic of each individual molecule, the substantial difference in the average transverse relaxation rate between the wild-type protein and the R62A mutant protein suggests that the substitution at residue Arg62 causes a subtle difference in rotational diffusion. The average R_2_/R_1_ value is 11.36 for wild-type and 12.02 for the R62A mutant and reflects the rotational correlation time of each protein. The slight increase in R_2_ in the R62A mutant, despite the almost identical in R_1_ values, implies that there might be motions on the microsecond-to-millisecond time scale induced by conformational exchange in the mutant protein that leads to line broadening. The order parameter S^2^ obtained from the isotropic model shows that the majority of the backbone amides are rigid, although the loops and the turns connecting the β-sheets show some dynamic behavior, and the N- and C-termini are highly flexible in both wild-type and R62A CheW (Fig. 4d). This conclusion is in agreement with a previous report [13]. Overall, the wild-type and mutant proteins had the same backbone dynamics.

In the model-free analysis, the phenomenological transverse relaxation rate constant, R_ex_, was found to make a significant contribution to achieving adequate fit of the ^15^N relaxation data during the calculations of the order parameter. This finding is in line with the previous results that suggest conformational motions in CheW that occur on the microsecond-to-millisecond time scale. For accurate characterization of the R_ex_ term, a series of Carr-Purcell-Meiboom-Gill (CPMG) 39–41 relaxation dispersion experiments were performed on both ^15^N labeled wild-type and R62A CheW [31], [37], [38]. The R_ex_ terms from R_1_/R_2_/NOE fitting were similar to the results from the individual CPMG measurements. The differences between R_ex_ measured at τ_cp_ values ranging from 20 ms to 1 ms for wild-type and R62A CheW are shown in Fig. 4e, and the differences between these two constructs are shown in Fig. 4f. In both proteins, the majority of the backbone ^15^N spins showed no significant differences in their relaxation rate constants. However, some residues located in β4 and β5, in loops, and in the two helical regions, showed relatively larger R_ex_ values in the mutant protein. Furthermore, the R62A change increased the R_ex_ value in some residues in loop1, α1, α2, β4 and β10, indicating that there are increased conformational-exchange motions on the microsecond-to-millisecond time scale in this region (Table S4). This finding suggests that the R62A substitution, which disrupts the interaction between Glu38 and Arg62, decreases the stability of the second subdomain of the CheW structure.

**Figure 5:**
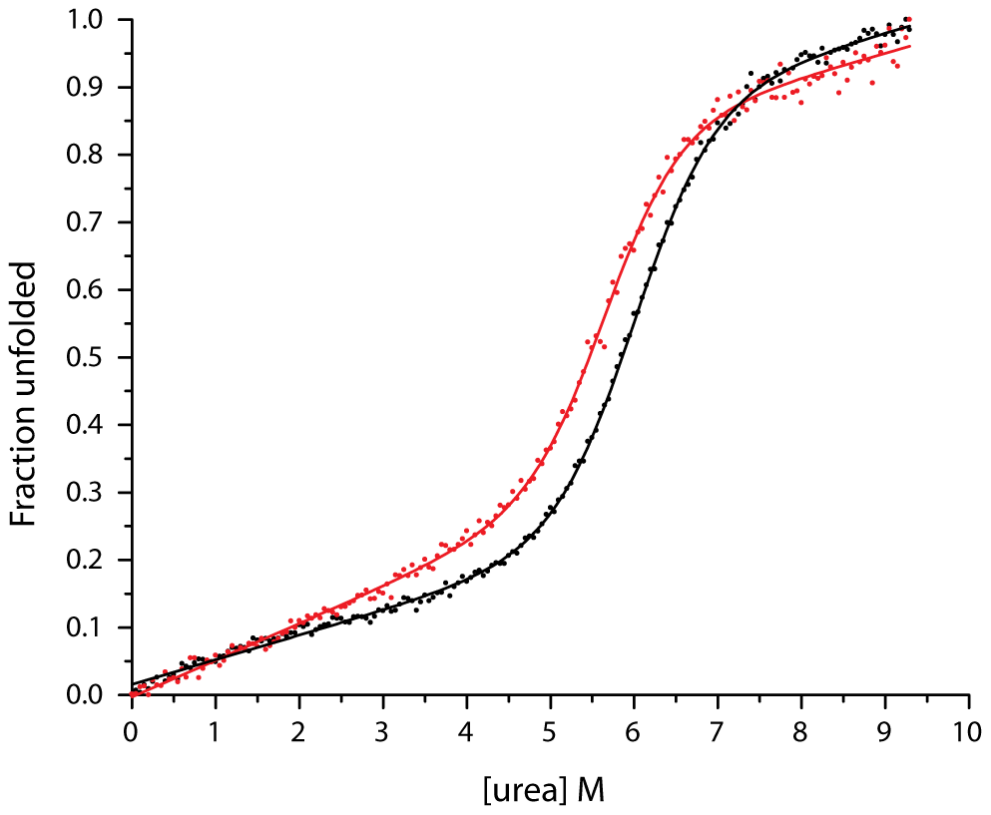
Urea-induced unfolding curves of far-UV CD spectra of CheW wild-type (black) and R62A mutant (red).

In addition, we detected no difference in the folding stability of the wild-type and R62A proteins in denaturation curves measured by circular dichroism as function of urea concentration (Fig. 5). The conformational stability of both variants was the same (Table 1). This result suggests that the Arg62 – Glu38 salt bridge does not influence overall protein stability and is not important in protein folding, in agreement with the results from the MD simulations and the NMR spectroscopy data. However, the difference in the slope of the pre-transition region of the unfolded fraction curves, m_f_, suggests that the Arg62-Glu38 bridge has a stabilizing effect when the protein is close to its native conformation (Fig. 5).

### Local backbone changes in Glu38 and Arg62 affect the relative position of chemoreceptor and kinase binding sites

**Figure 6:**
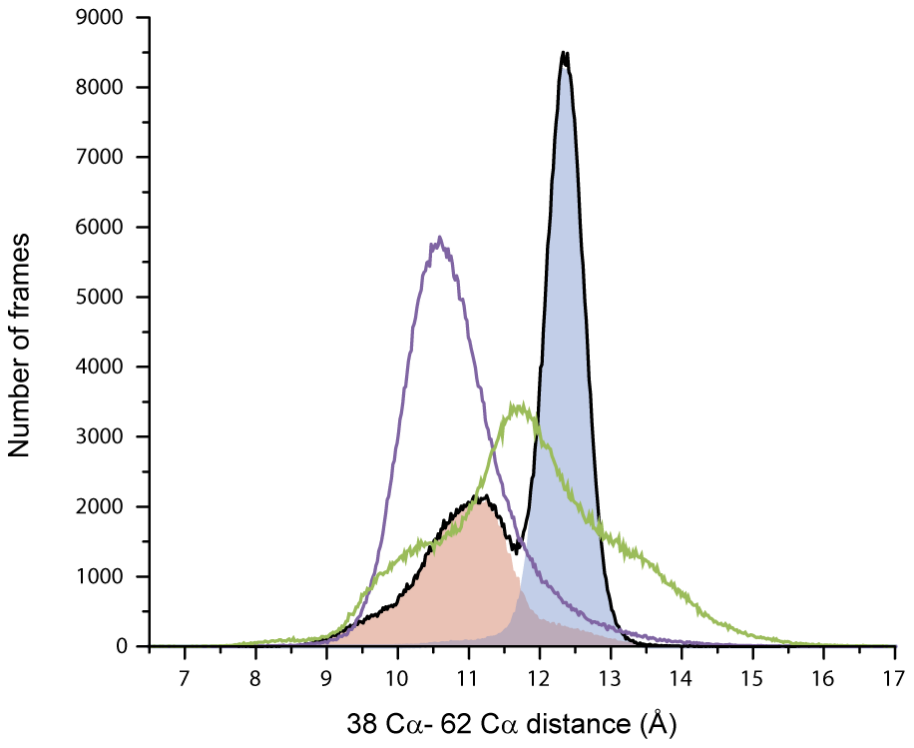
Histogram of the distance between alpha carbon from residues in position 38 and 62 in all simulations of wild-type (black), E38A (purple) and R62A (green). The frames of the wild-type simulations can be separated by the occurrence of salt-bridges in geometry A (blue shade) and all other interactions (red shade). The latter includes frames with geometry B and frames with no interaction between the residues Arg62 and Glu38. The salt-bridge in geometry A is solely responsible for the peak of stability of alpha carbon distance in 12.3±0.3 Å. Both mutants show an increase in instability (broader peaks) with respect to the wild-type salt-bridge with geometry A (blue shade).

To understand the contribution of the salt bridge to local backbone stability in more detail, we analyzed the distance between alpha carbons of all relevant residues in 10 MD simulations. We found that the salt-bridge in geometry A maintains the distance between the alpha carbons of residues Glu38 and Arg62 at 12.3±0.3 Å. All other conformations assumed by the ion pair, including the salt-bridge in geometry B and the N-O bridge, slightly shift the distance between the alpha carbons and increase their relative motion range (Fig. 6). The independent distribution of each conformation is shown on Fig. S4. This result indicates that the maintenance of the correct distance between the backbone atoms of Glu38 and Arg62 is compromised if the residues do not form a salt-bridge in geometry A. To further validate this finding, we carried out ten independent simulations of 90 ns each for the *in silico* E38A and R62A proteins. Both mutations intrinsically forbid salt-bridge formation. In both mutant proteins, the distance between the backbone atoms, and specifically alpha carbons, was not restricted, as it was in the wild-type protein with the salt-bridge in geometry A (Fig. 6). Overall, shifts in distances between alpha carbons (Fig. 6 and Fig. S4) were significant (>1.5 Å). For example, a helical displacement of only 2 Å initiates the signaling cascade in the transmembrane chemoreceptors [60]–[62].Thus, we conclude that the formation of the salt-bridge between residues Glu38 and Arg62 in a specific geometry maintains the positions of their corresponding backbone atoms in a stable relationship.

Arg62 is located close to a proposed CheA-binding site, and Glu38 is located within a proposed chemoreceptor-binding site. Consistent changes in alpha carbon fluctuations calculated for each variant show an increase in the motion of the chemoreceptor-binding site relative to the kinase-binding site. Local changes in backbone positions relative to these sites were seen in all frames in which the interaction between Arg62 and Glu38 was not maintained in geometry A. As revealed by the analyses of the order parameter derived from the molecular dynamics simulations, and in agreement with the values calculated in NMR studies, this local change in backbone mobility is not a result of changes in overall protein dynamics in the pico-to-nanosecond time scale (Fig. S5).

**Figure 7:**
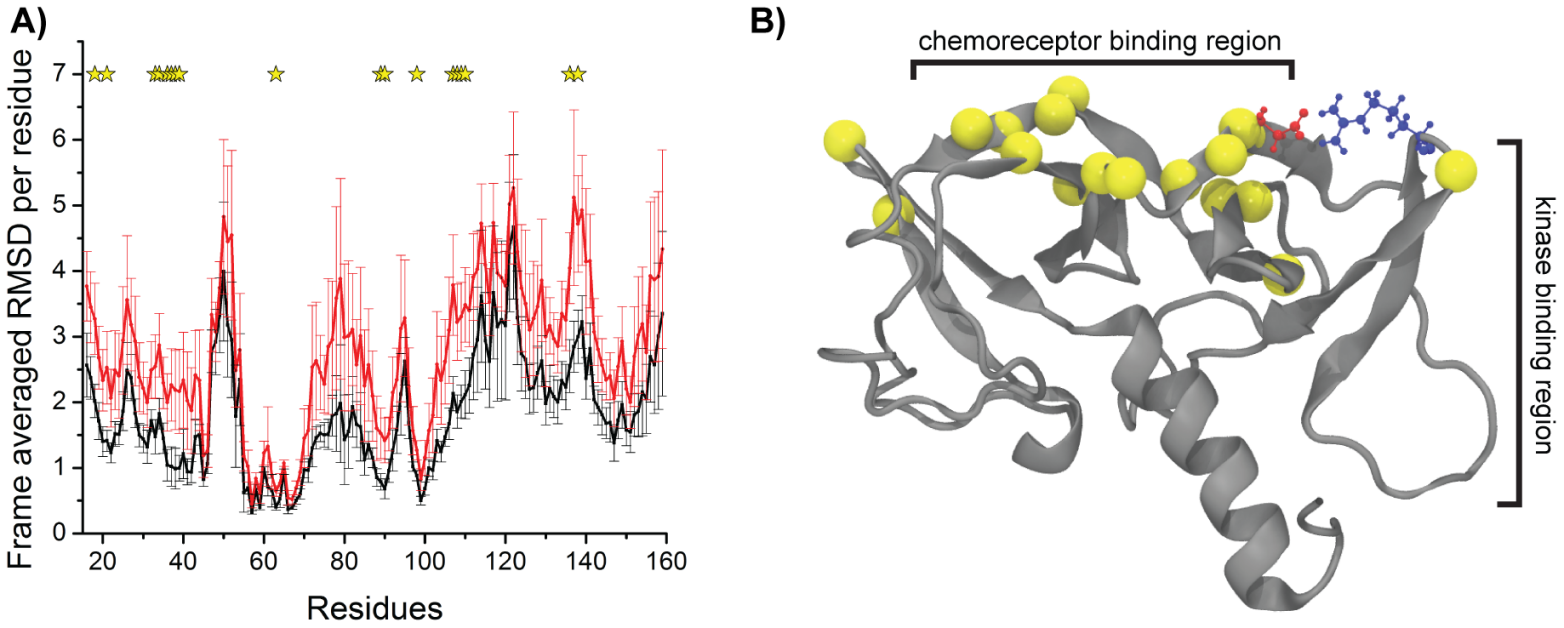
Salt bridge in geometry A between Arg62 and Glu38 improves the stability of the position of the chemoreceptor binding region relative to the kinase binding region. (A) Mean value of the frame-averaged root mean square deviation (RMSD) per residue calculated for each wild-type simulation with salt-bridge in geometry A between Arg62 and Glu38 (black) and also for each R62A simulations (red). The error bars represent standard error of the mean. (B) Cartoon representation of the CheW structure and residues Arg62 (blue) and Glu38 (red). Residues presenting significant difference (P<0.0002) in RMSD are marked in the plot (yellow star) and mapped in the structure (yellow spheres).

To examine the consequences of salt bridge disruption on the chemoreceptor- and kinase-binding sites, we analyzed the difference in frame-averaged root mean square deviation (RMSD) per residue between frames collected from the simulations of the R62A protein in comparison with those of the wild-type protein, with the salt bridge in geometry A. (The Ala substitution at Glu38 disrupts the interaction of CheW with the receptor [23], so we did not perform the same analysis for the E38A protein). To measure the fluctuation of the chemoreceptor-binding region relative to the CheA-binding region, we aligned the frames using only the backbone atoms of residues Ile55 to Val68, which is a proposed CheA-binding site [12], [23]. The frames with the salt bridge in geometry A were selected separately from each simulation, and the final frame-averaged RMSD per residue value is an average of the values independently calculated for each simulation. Because only 64% of the frames from the wild-type simulation had a salt bridge in geometry A, we randomly selected 64% of the frames from all ten R62A simulations. Overall, the R62A mutant protein was more dynamic than the wild-type (higher frame-averaged RMSD per residue) (Fig. 7A). However, considering the fluctuation of the results from simulation to simulation, only a few residues were significantly more dynamic in the R62A protein than in the wild-type protein (p-value<0.00002) (Table S5). More than half of these residues were found in the chemoreceptor-binding region (Fig. 7B), a result which further supports our hypothesis. Taken together, these results suggest that the most important consequence of disrupting the salt bridge between Glu38 and Arg62 is an increase in fluctuation of the relative positions between the kinase and receptor binding sites on the CheW surface.

## Discussion

The results presented here provide a compelling explanation for the strong evolutionary pressure on residues Arg62 and Glu38 of the chemoreceptor scaffold protein CheW. These residues are invariant in all currently available CheW sequences from the most populated chemotaxis class F7, which contains chemotaxis systems of representatives of diverse bacterial phyla [44]. Glu38 was previously suggested to participate in the interaction with chemoreceptors [23], however, earlier studies failed to propose a specific role for Arg62 despite the fact that this residue was recognized as conserved and shown to be critical for chemotaxis [24]. Using MD simulations, we demonstrated that Arg62 and Glu38 can form a stable salt-bridge with a specific geometry. This result could not be obtained using any other experimental method, such as pH titration, given the dynamic properties of the CheW protein, which precipitates in pH values lower than 6.0. Simulations with the R62A mutant show that disruption of the salt bridge does not compromise the overall structure or dynamics of the protein. However, it results in a detectable loss in maintaining a stable relationship between the chemoreceptor- and the kinase-binding regions. NMR experiments showed that the R62A substitution only perturbs the CheW structure locally, in agreement with the MD results. In contrast, the chemical shifts of several important residues in the E38A mutant protein indicate that this position is important for the structural integrity of at least one (C-terminal) subdomain of CheW. Furthermore, the NMR relaxation-dispersion experiment suggests that there are local motions on microsecond-to-millisecond time scale in the R62A mutant. The increase in the rotational correlation time for the R62A mutant protein suggests that this substitution may lead to an overall subtle expansion of the molecule.

Taken together, we propose that motions in pico-to-nanoseconds time scale explicitly caused by the disruption of the Glu38-Arg62 as observed in the MD simulations are likely to cause subtle changes in the protein structure without changing the overall protein stability. These changes are likely to allow new vibration modes in the microsecond-to-millisecond time scale with larger excursions than encountered in the wild-type protein, as suggested by the increase in the rotational correlation time. In addition, the analysis of urea-induced unfolding curves showed that disruption of the salt bridge does not affect the conformational stability of the protein. However, the difference in the slope of the pre-transition part of the curve of the wild-type and the R62A variant proteins suggests that the interaction between Arg62 and Glu38 provides a stabilizing role in the near native-protein conformations. Finally, there was a good agreement between the NMR and MD measurements of the order parameter for both the R62A and the wild-type proteins, which provided experimental validation of computer simulations.

Our results show that the major structural difference in the R62A mutant is the destabilization of the relative position of the chemoreceptor-binding site relative to the kinase-binding site. On a larger scale, this translates into a relaxation of the precise orientation of the chemoreceptor relative to the kinase. Thus, the Glu38-Arg62 bridge is stabilizing, i.e., it constrains flexibility and motion as do most of salt bridges [63]. We conclude that, although this salt bridge is not required for assembly of the signaling complex, it “tightens” the elements of the complex together thus enabling signal transduction.

The stabilization provided by the Glu38-Arg62 salt-bridge appears to be required for chemotaxis. The retention of this feature in the entire chemotaxis class F7 attests to its importance. It is present in the CheW proteins in such important pathogens as *Bordetella bronchiseptica* (BB2547, CheW locus tag), *Clostridium difficile* (CD0536), *Salmonella enterica* (t0957), *Pseudomonas aeruginosa* (PA0177), *Vibrio cholerae* (VCA1094), *Yersinia pestis* (YPO1667) and many others. However, whether or not the same mechanism is utilized in CheW proteins from other chemotaxis classes is unclear. For example, analysis of the crystal structure of the CheW protein (TTE0700, locus tag) from of *Thermoanaerobacter tengcongensis* (class F1) reveals the same Glu38-Arg62 salt-bridge (residues Glu33 and Arg57 in the TTE0700 sequence) [14]. However, neither of the two CheW proteins from *Thermotoga maritima* [64] (both from class F1) have the conserved glutamate at the same position. Analysis of the crystal structures [65], [66] and the NMR model [10] of *T. maritima* CheW2 (TM0701, locus tag) suggests a similar mechanism through a salt-bridge formed by another Glu-Arg pair (Glu31 and Arg58 in the TM0701 sequence). Thus, CheW proteins from other chemotaxis classes might utilize different amino acid positions for the same strategy - stabilizing the relative position between the chemoreceptor- and the kinase-binding sites.

Recent advances in determining crystal structures of interacting CheW and CheA proteins and electron cryotomography of signaling arrays have provided static models of the entire chemotaxis signaling complex [65], [67]. However, protein interactions are dynamic, not static. In recent years, a number of studies have aimed at improving our understanding of protein-protein interactions and their roles in biological processes by revealing their evolution and dynamic properties. (For a review, see [68]). Our study builds on these recent developments. We still do not know the molecular mechanisms of signal transduction in the signaling complex, and studies of protein dynamics will provide a complement to other avenues of current research in chemotaxis. CheW appears to be a rapidly evolving and highly dynamic protein. These two features usually correlate: Properties that make proteins conformationally dynamic also facilitate rapid evolution [69]. CheW proteins from different prokaryotes share very little sequence similarity [55], in striking contrast to their interacting partners - chemoreceptors and CheA [44], [54]. Likewise, low sequence similarity and high diversification are observed in SH3 domains from eukaryotic scaffold proteins [16], [70] that are topologically similar to CheW [15]. Therefore, the high conformational dynamics coupled with stabilization mechanisms of the type discussed here for CheW may be important universal properties of scaffold proteins that participate in assembling arrays of proteins involved in signal transduction.

## Supporting Information

Figure S1 Distribution of non-redundant CheW sequences in chemotaxis classes.
(TIF)

Figure S2 Temporal evolution of the distance of the side-chain charged group centroids.
(TIF)

Figure S3 Effects of the mutation E38A in the CheW structure. A) Superposition of ^1^H-^15^N HSQC spectra of wild-type CheW (black) and the mutant CheW E38A (red). B) The chemical shift perturbation between wild-type- and E38A CheW color-mapped onto the CheW structure (PDB code 2HO9). The red color indicates larger chemical shift difference and blue color showed smaller differences. The mutation site E38 is shown in yellow.
(TIF)

Figure S4 Distributions of the distances between alpha carbons for each conformation between Arg62 and Glu38 in all simulations with wild-type. In black is the sum of all conformations, salt bridge in geometry A is in blue, salt bridge in geometry B is in red, salt bridge in other salt-bridge geometries is in purple, N-O bridge in light green and finally longer range in dark green. Note the log scale for easy display of the less populated conformations.
(TIF)

Figure S5 Average of the order parameter calculations for 10 simulations in each simulated allele. Wild-type is in black, E38A in red and R62A in blue. Only local changes around position 62 and loop regions are prone of consistent changes in dynamics. Error bars represent the standard deviation of the calculations for the 10 simulation in each allele.
(TIF)

Table S1 Circular dichroism data collected for the wild-type and R62A CheW variants.
(PDF)

Table S2 Top 5 most conserved residues in F1 and F7 classes of the CheW protein.
(PDF)

Table S3 ^15^N HSQC Chemical Shift assignment in 30C° for the wild-type, R62A and E38A alleles. Buffer: 30 mM Tris-HCl (pH 7.3), 30 mM NaCl, 0.2% sodium azide in 90% H_2_O and 10% D_2_O.
(PDF)

Table S4 Differences phenomenological transverse relaxation rate constant ΔR_ex_ = R_ex_(20 ms)−R_ex_(1 ms) and ΔΔRex the differences between the ΔR_ex_ in R62A and wild-type. Residues with large ΔΔR_ex_ in bold are likely to be involved in motions in the microsecond-to-millisecond time scale.
(PDF)

Table S5 Root mean square deviation per residue of all residues for each simulation. Frames were aligned using backbone atoms of the residues Ile55 to Val68, which is proposed to be the CheA binding site. We use T-test to find RMSD per residue values significantly different from wild-type and R62A simulations. Values with p-value<0.00002 are shown in bold.
(PDF)
