# Supplementary figures and images for: Conformational Coupling between Receptor and Kinase Binding Sites through a Conserved Salt Bridge in a Signaling Complex Scaffold Protein
Source: PLoS Comput Biol. 2013 Nov 14;9(11):e1003337. doi: 10.1371/journal.pcbi.1003337 (PMC3828127; doi:10.1371/journal.pcbi.1003337)

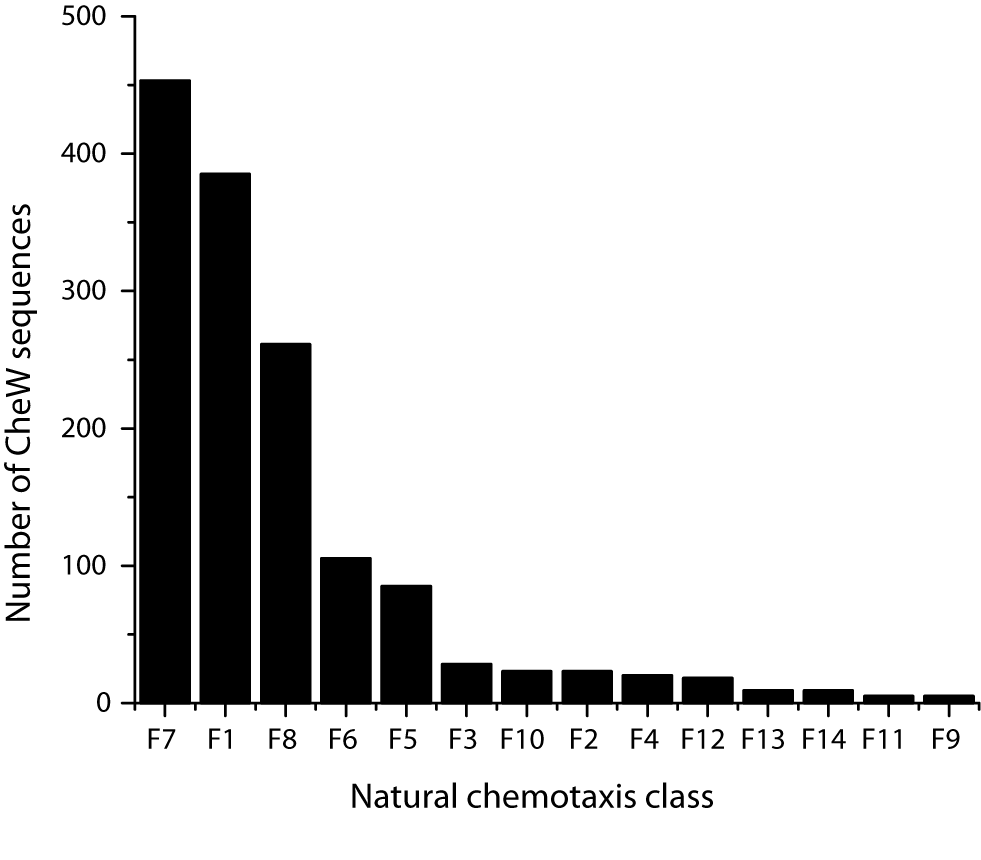

Supplement: Figure S1 — Distribution of non-redundant CheW sequences in chemotaxis classes. (TIF) [file pcbi.1003337.s001.tif]

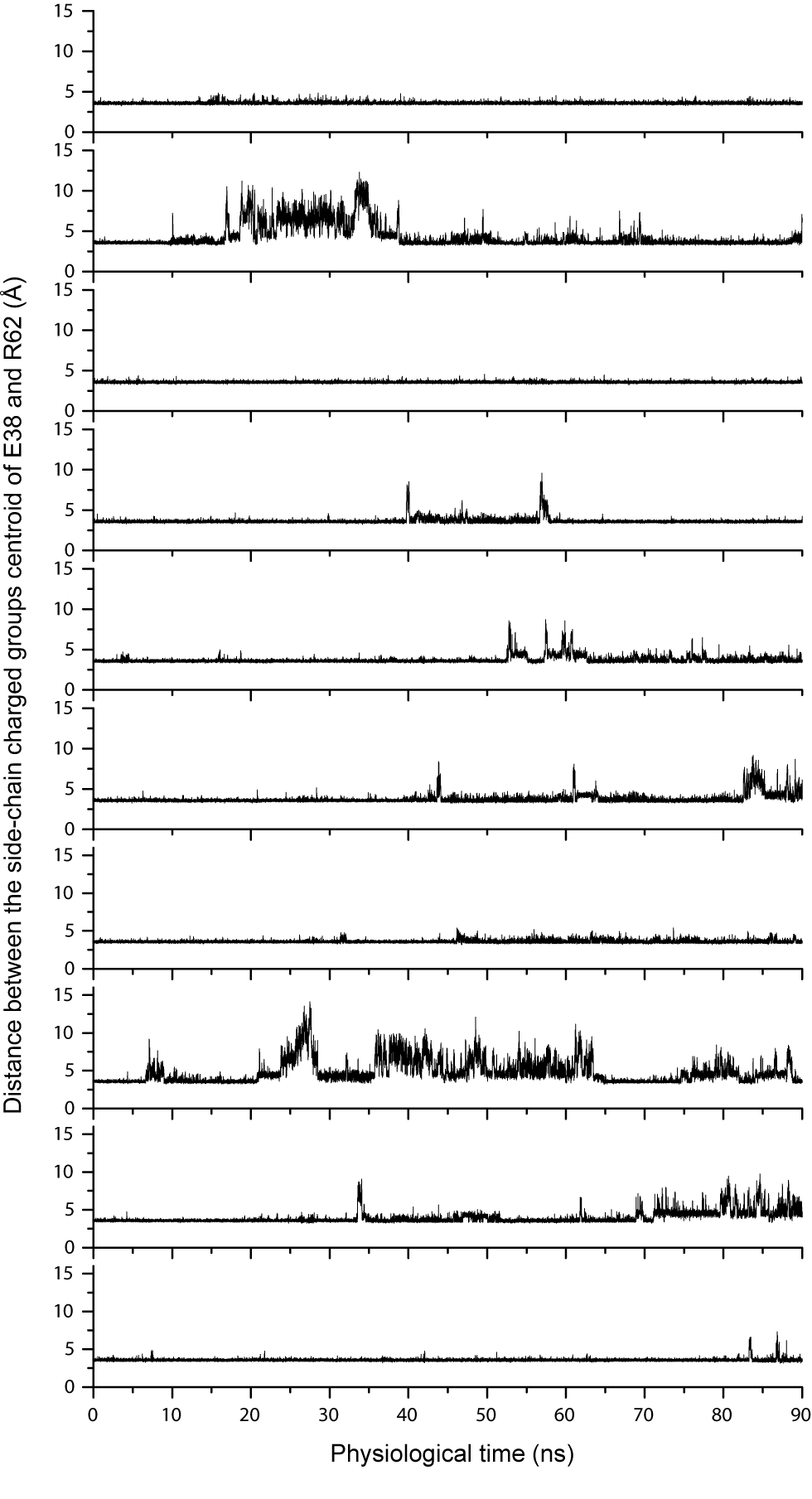

Supplement: Figure S2 — Temporal evolution of the distance of the side-chain charged group centroids. (TIF) [file pcbi.1003337.s002.tif]

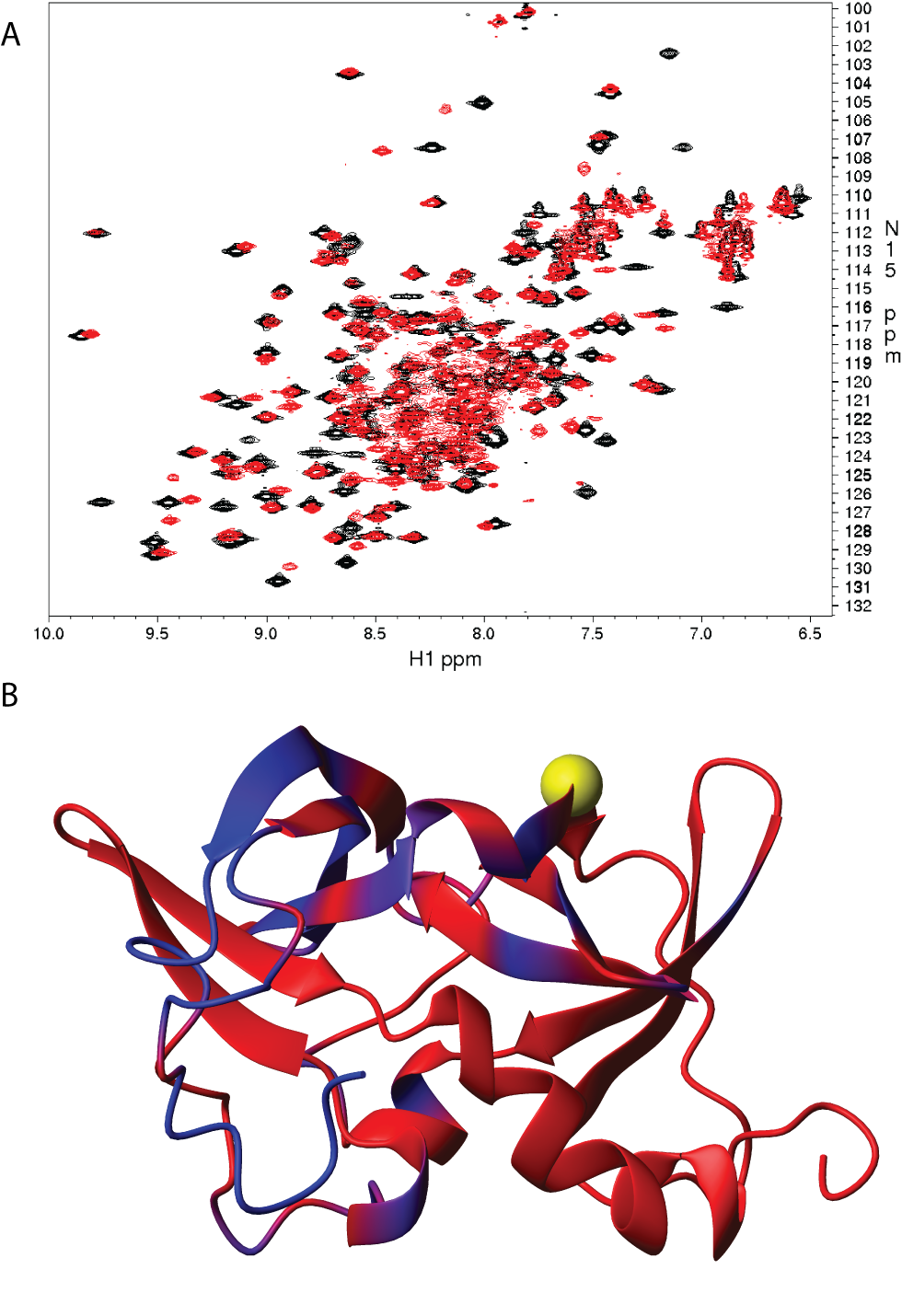

Supplement: Figure S3 — Effects of the mutation E38A in the CheW structure. A) Superposition of 1H-15N HSQC spectra of wild-type CheW (black) and the mutant CheW E38A (red). B) The chemical shift perturbation between wild-type- and E38A CheW color-mapped onto the CheW structure (PDB code 2HO9). The red color indicates larger chemical shift difference and blue color showed smaller differences. The mutation site E38 is shown in yellow. (TIF) [file pcbi.1003337.s003.tif]

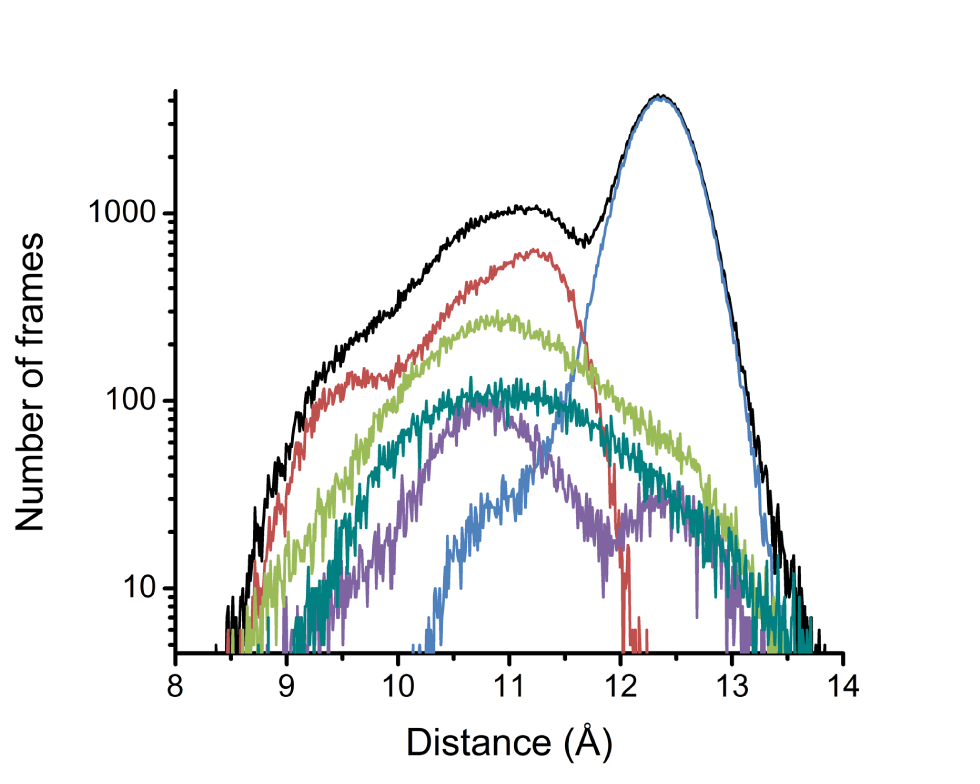

Supplement: Figure S4 — Distributions of the distances between alpha carbons for each conformation between Arg62 and Glu38 in all simulations with wild-type. In black is the sum of all conformations, salt bridge in geometry A is in blue, salt bridge in geometry B is in red, salt bridge in other salt-bridge geometries is in purple, N-O bridge in light green and finally longer range in dark green. Note the log scale for easy display of the less populated conformations. (TIF) [file pcbi.1003337.s004.tif]

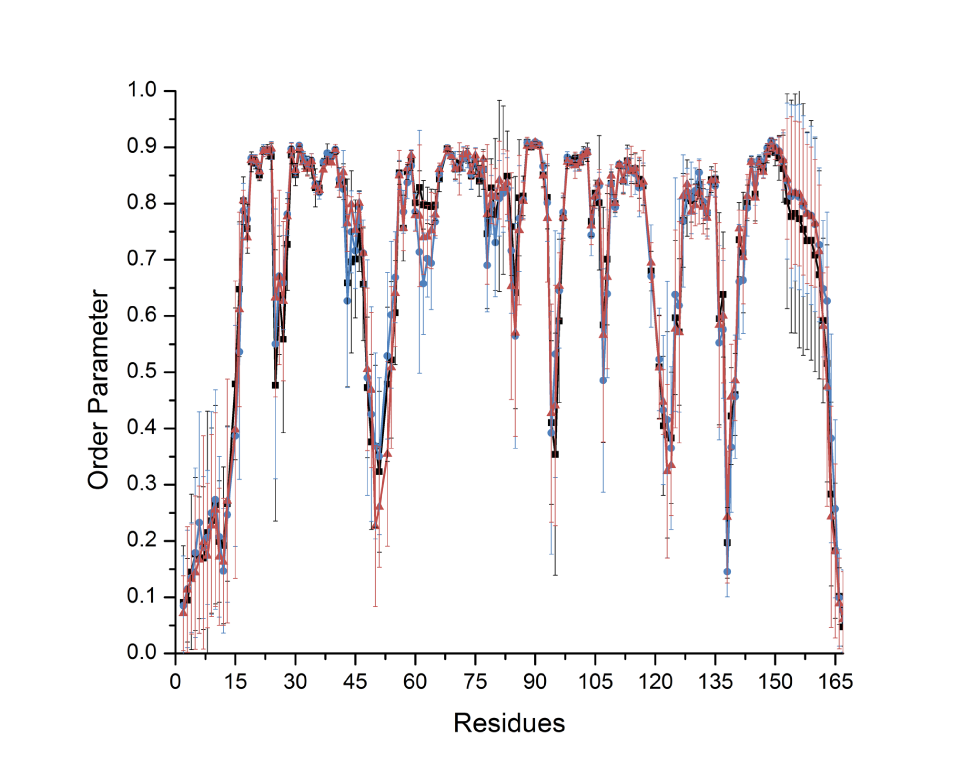

Supplement: Figure S5 — Average of the order parameter calculations for 10 simulations in each simulated allele. Wild-type is in black, E38A in red and R62A in blue. Only local changes around position 62 and loop regions are prone of consistent changes in dynamics. Error bars represent the standard deviation of the calculations for the 10 simulation in each allele. (TIF) [file pcbi.1003337.s005.tif]
