# Supplementary material for: Conformational Coupling between Receptor and Kinase Binding Sites through a Conserved Salt Bridge in a Signaling Complex Scaffold Protein
Source: PLoS Comput Biol. 2013 Nov 14;9(11):e1003337. doi: 10.1371/journal.pcbi.1003337 (PMC3828127; doi:10.1371/journal.pcbi.1003337)
Supplement: Table S1 — Circular dichroism data collected for the wild-type and R62A CheW variants. (PDF) [file pcbi.1003337.s006.pdf]

Table S1: Circular dichroism data collected for the wild-type and R62A CheW variants.

	CD signal @ 220 nm						Average	
[Urea] (M)	Wild type #1	Wild type #2	Wild type #3	Wild type #4	R62A #1	R62A #2	Wild type	R62A
0	-82.282	-79.425	-85.994	-80.530	-65.941	-72.077	-82.05775	-69.009
0.05	-82.615	-76.993	-85.662	-81.388	-66.194	-71.781	-81.6645	-68.9875
0.1	-81.743	-78.757	-86.330	-80.559	-65.507	-71.398	-81.84725	-68.4525
0.15	-81.798	-78.064	-85.184	-79.750	-65.341	-71.468	-81.199	-68.4045
0.2	-81.421	-77.627	-86.412	-80.638	-66.262	-71.865	-81.5245	-69.0635
0.25	-81.552	-76.448	-85.569	-80.074	-64.732	-71.124	-80.91075	-67.928
0.3	-81.289	-76.885	-84.410	-79.517	-65.2	-71.425	-80.52525	-68.3125
0.35	-81.595	-76.887	-85.999	-80.556	-64.813	-71.900	-81.25925	-68.3565
0.4	-81.451	-77.086	-84.679	-80.230	-64.536	-70.077	-80.8615	-67.3065
0.45	-80.910	-76.568	-84.745	-79.998	-64.749	-71.015	-80.55525	-67.882
0.5	-81.606	-76.124	-83.690	-79.767	-64.582	-70.941	-80.29675	-67.7615
0.55	-80.829	-76.078	-85.307	-80.546	-65.227	-70.917	-80.69	-68.072
0.6	-80.477	-75.259	-83.138	-78.425	-64.077	-70.095	-79.32475	-67.086
0.65	-80.395	-75.193	-83.420	-79.227	-64.79	-70.652	-79.55875	-67.721
0.7	-81.142	-74.579	-83.526	-79.529	-63.211	-69.231	-79.694	-66.221
0.75	-81.277	-75.070	-82.627	-78.108	-62.941	-69.548	-79.2705	-66.2445
0.8	-80.578	-74.392	-82.464	-78.335	-64.554	-70.948	-78.94225	-67.751
0.85	-80.335	-74.098	-81.487	-79.991	-63.927	-70.165	-78.97775	-67.046
0.9	-81.338	-74.238	-81.958	-78.634	-63.425	-69.370	-79.042	-66.3975
0.95	-80.267	-75.299	-82.390	-78.667	-63.037	-70.057	-79.15575	-66.547
1	-80.145	-74.614	-82.478	-79.477	-62.915	-69.166	-79.1785	-66.0405
1.05	-80.597	-74.129	-81.832	-78.246	-64.124	-69.532	-78.701	-66.828
1.1	-80.517	-75.051	-81.666	-77.454	-63.24	-69.655	-78.672	-66.4475
1.15	-79.661	-74.008	-81.939	-77.420	-63.093	-68.420	-78.257	-65.7565
1.2	-79.476	-74.885	-81.073	-76.286	-62.377	-68.223	-77.93	-65.3
1.25	-79.547	-74.091	-80.539	-77.887	-62.741	-68.802	-78.016	-65.7715
1.3	-79.669	-73.044	-80.941	-77.640	-63.227	-67.684	-77.8235	-65.4555
1.35	-78.864	-75.030	-79.512	-78.358	-62.417	-68.709	-77.941	-65.563
1.4	-80.985	-73.505	-81.908	-76.526	-62.948	-67.331	-78.231	-65.1395
1.45	-78.503	-74.120	-79.948	-75.845	-62.502	-67.813	-77.104	-65.1575
1.5	-79.397	-74.428	-79.739	-75.577	-62.283	-68.426	-77.28525	-65.3545
1.55	-79.526	-73.852	-79.670	-76.422	-62.154	-67.318	-77.3675	-64.736
1.6	-78.511	-74.083	-79.426	-76.558	-62.709	-68.459	-77.1445	-65.584
1.65	-78.682	-73.624	-80.193	-77.000	-62.648	-67.958	-77.37475	-65.303
1.7	-78.403	-73.465	-79.862	-77.005	-61.604	-68.433	-77.18375	-65.0185
1.75	-78.813	-73.494	-79.085	-77.034	-61.607	-67.897	-77.1065	-64.752
1.8	-78.756	-73.625	-79.240	-75.282	-61.713	-67.049	-76.72575	-64.381
1.85	-78.352	-74.128	-79.152	-76.187	-60.663	-67.816	-76.95475	-64.2395
1.9	-78.672	-73.207	-78.647	-76.066	-61.169	-65.620	-76.648	-63.3945
1.95	-77.830	-72.859	-79.335	-76.565	-61.835	-65.947	-76.64725	-63.891
2	-77.487	-72.156	-78.749	-75.373	-60.493	-66.270	-75.94125	-63.3815
2.05	-77.555	-72.994	-78.318	-75.575	-60.852	-66.267	-76.1105	-63.5595
2.1	-77.516	-71.967	-78.043	-76.095	-59.644	-66.404	-75.90525	-63.024
2.15	-78.058	-73.340	-79.059	-76.792	-60.242	-66.146	-76.81225	-63.194
2.2	-78.291	-72.613	-79.443	-75.138	-60.4	-65.488	-76.37125	-62.944
2.25	-77.255	-72.076	-79.724	-75.309	-60.426	-65.938	-76.091	-63.182

2.3	-78.145	-72.407	-78.067	-75.115	-60.07	-64.900	-75.9335	-62.485
2.35	-77.126	-72.161	-78.355	-75.919	-60.155	-65.080	-75.89025	-62.6175
2.4	-77.188	-72.876	-77.264	-73.860	-60.5	-64.987	-75.297	-62.7435
2.45	-77.459	-71.515	-76.982	-75.599	-59.581	-66.280	-75.38875	-62.9305
2.5	-77.399	-72.601	-76.663	-74.827	-60.478	-64.152	-75.3725	-62.315
2.55	-77.439	-72.492	-78.329	-74.518	-59.769	-64.803	-75.6945	-62.286
2.6	-77.350	-73.215	-77.909	-74.552	-59.812	-64.261	-75.7565	-62.0365
2.65	-77.108	-72.406	-77.119	-74.364	-60.092	-63.658	-75.24925	-61.875
2.7	-76.817	-72.200	-77.931	-73.830	-59.713	-63.215	-75.1945	-61.464
2.75	-76.451	-73.012	-76.751	-74.750	-59.435	-63.427	-75.241	-61.431
2.8	-76.537	-72.987	-78.197	-73.778	-58.581	-63.525	-75.37475	-61.053
2.85	-77.382	-72.947	-77.666	-74.927	-59.109	-64.293	-75.7305	-61.701
2.9	-75.745	-72.136	-75.889	-77.071	-59.048	-64.350	-75.21025	-61.699
2.95	-76.428	-72.381	-76.015	-73.729	-59.605	-62.731	-74.63825	-61.168
3	-76.170	-71.626	-77.255	-73.835	-58.305	-64.250	-74.7215	-61.2775
3.05	-75.907	-71.759	-75.873	-73.573	-58.249	-62.925	-74.278	-60.587
3.1	-76.069	-72.911	-76.212	-73.981	-59.237	-63.998	-74.79325	-61.6175
3.15	-76.579	-71.834	-76.219	-74.013	-57.124	-62.624	-74.66125	-59.874
3.2	-75.845	-71.589	-75.661	-73.522	-57.658	-62.206	-74.15425	-59.932
3.25	-76.138	-70.623	-75.317	-72.267	-57.22	-61.728	-73.58625	-59.474
3.3	-74.489	-69.986	-76.655	-73.490	-57.836	-62.018	-73.655	-59.927
3.35	-76.575	-70.161	-76.375	-72.333	-57.121	-61.145	-73.861	-59.133
3.4	-76.651	-70.508	-76.977	-74.579	-58.573	-61.178	-74.67875	-59.8755
3.45	-75.669	-70.250	-74.970	-74.870	-57.521	-61.131	-73.93975	-59.326
3.5	-75.213	-70.986	-74.236	-73.001	-57.068	-60.275	-73.359	-58.6715
3.55	-74.943	-69.916	-75.762	-74.770	-57.753	-60.840	-73.84775	-59.2965
3.6	-75.416	-70.683	-74.643	-73.511	-57.738	-61.083	-73.56325	-59.4105
3.65	-74.818	-70.999	-73.760	-72.974	-55.613	-61.247	-73.13775	-58.43
3.7	-75.049	-70.365	-73.914	-73.132	-55.202	-59.915	-73.115	-57.5585
3.75	-73.526	-70.614	-74.502	-70.515	-56.234	-59.053	-72.28925	-57.6435
3.8	-74.262	-71.238	-74.391	-73.713	-56.465	-59.452	-73.401	-57.9585
3.85	-73.417	-70.233	-74.366	-72.440	-55.574	-60.249	-72.614	-57.9115
3.9	-73.899	-69.204	-73.823	-72.167	-55.838	-59.243	-72.27325	-57.5405
3.95	-72.249	-69.885	-73.552	-71.197	-55.389	-58.830	-71.72075	-57.1095
4	-73.913	-69.812	-73.955	-70.936	-54.603	-58.383	-72.154	-56.493
4.05	-72.813	-68.743	-73.152	-72.514	-55.744	-59.324	-71.8055	-57.534
4.1	-72.741	-69.841	-70.423	-72.440	-55.948	-57.670	-71.36125	-56.809
4.15	-71.885	-69.405	-71.789	-71.634	-53.936	-57.519	-71.17825	-55.7275
4.2	-72.893	-70.249	-72.223	-71.277	-54.478	-58.808	-71.6605	-56.643
4.25	-73.190	-68.712	-71.934	-71.142	-53.314	-58.384	-71.2445	-55.849
4.3	-70.928	-68.788	-71.643	-71.597	-53.825	-58.400	-70.739	-56.1125
4.35	-70.820	-69.512	-72.037	-69.669	-53.825	-56.896	-70.5095	-55.3605
4.4	-70.839	-69.151	-71.805	-70.509	-53.28	-55.781	-70.576	-54.5305
4.45	-71.245	-69.707	-71.294	-70.145	-53.499	-55.914	-70.59775	-54.7065
4.5	-71.018	-69.458	-70.279	-68.787	-52.173	-56.861	-69.8855	-54.517
4.55	-70.968	-68.018	-69.596	-69.667	-51.981	-55.024	-69.56225	-53.5025
4.6	-70.796	-68.357	-70.567	-68.991	-51.506	-56.542	-69.67775	-54.024
4.65	-69.206	-66.965	-70.823	-69.167	-50.471	-54.822	-69.04025	-52.6465
4.7	-68.314	-67.727	-68.183	-69.221	-50.916	-55.691	-68.36125	-53.3035
4.75	-66.701	-68.078	-70.372	-67.675	-52.12	-53.271	-68.2065	-52.6955

4.8	-69.039	-69.639	-68.067	-66.613	-50.941	-54.036	-68.3395	-52.4885
4.85	-67.403	-67.201	-68.134	-68.305	-49.587	-52.636	-67.76075	-51.1115
4.9	-66.849	-66.063	-68.582	-67.053	-49.082	-53.653	-67.13675	-51.3675
4.95	-64.652	-65.756	-68.134	-66.688	-48.691	-51.947	-66.3075	-50.319
5	-67.542	-65.000	-65.994	-64.231	-48.689	-51.662	-65.69175	-50.1755
5.05	-66.146	-65.549	-66.256	-66.337	-47.967	-51.371	-66.072	-49.669
5.1	-64.489	-64.596	-65.248	-65.860	-46.923	-49.722	-65.04825	-48.3225
5.15	-63.861	-64.529	-65.542	-64.992	-46.771	-47.978	-64.731	-47.3745
5.2	-64.738	-63.118	-64.914	-63.435	-46.339	-49.050	-64.05125	-47.6945
5.25	-62.852	-64.504	-63.439	-63.453	-46.539	-47.811	-63.562	-47.175
5.3	-61.173	-62.196	-63.747	-61.040	-45.163	-47.830	-62.039	-46.4965
5.35	-61.723	-62.321	-61.189	-61.412	-44.557	-45.731	-61.66125	-45.144
5.4	-60.269	-61.727	-61.974	-62.545	-43.703	-44.921	-61.62875	-44.312
5.45	-59.497	-60.223	-60.021	-59.872	-40.665	-43.374	-59.90325	-42.0195
5.5	-58.243	-59.438	-59.560	-61.134	-41.566	-43.389	-59.59375	-42.4775
5.55	-56.517	-58.778	-60.404	-60.086	-40.126	-43.006	-58.94625	-41.566
5.6	-56.034	-58.218	-58.209	-57.467	-40.618	-43.405	-57.482	-42.0115
5.65	-56.133	-56.977	-56.262	-57.618	-41.524	-43.278	-56.7475	-42.401
5.7	-54.619	-56.695	-55.374	-58.184	-38.478	-39.279	-56.218	-38.8785
5.75	-53.344	-54.439	-54.785	-56.080	-36.314	-38.594	-54.662	-37.454
5.8	-51.886	-53.999	-52.900	-54.821	-36.605	-39.854	-53.4015	-38.2295
5.85	-50.437	-53.167	-50.403	-55.359	-34.671	-36.296	-52.3415	-35.4835
5.9	-48.094	-51.882	-51.130	-53.031	-33.307	-36.415	-51.03425	-34.861
5.95	-48.708	-50.678	-50.975	-52.314	-33.143	-35.856	-50.66875	-34.4995
6	-46.711	-49.793	-48.487	-50.051	-33.971	-36.033	-48.7605	-35.002
6.05	-48.063	-50.100	-46.628	-49.643	-32.821	-34.393	-48.6085	-33.607
6.1	-45.867	-47.386	-46.974	-49.171	-32.298	-34.397	-47.3495	-33.3475
6.15	-43.484	-46.123	-46.567	-48.844	-29.354	-33.569	-46.2545	-31.4615
6.2	-43.624	-46.372	-43.396	-46.185	-31.548	-33.111	-44.89425	-32.3295
6.25	-42.850	-45.200	-44.908	-46.455	-29.825	-31.756	-44.85325	-30.7905
6.3	-41.817	-43.331	-41.980	-43.881	-28.07	-30.733	-42.75225	-29.4015
6.35	-40.318	-44.182	-41.777	-43.369	-29.086	-31.985	-42.4115	-30.5355
6.4	-39.221	-41.422	-41.128	-41.492	-27.332	-28.464	-40.81575	-27.898
6.45	-39.010	-39.640	-40.733	-44.017	-28.382	-29.435	-40.85	-28.9085
6.5	-39.148	-39.346	-36.695	-42.410	-26.944	-29.039	-39.39975	-27.9915
6.55	-38.527	-38.245	-38.405	-40.168	-26.453	-28.849	-38.83625	-27.651
6.6	-36.718	-39.851	-36.479	-38.653	-24.908	-28.128	-37.92525	-26.518
6.65	-36.200	-37.264	-35.301	-41.182	-25.981	-27.079	-37.48675	-26.53
6.7	-35.189	-37.250	-36.508	-38.456	-25.674	-27.907	-36.85075	-26.7905
6.75	-34.382	-34.189	-35.316	-37.256	-25.463	-27.349	-35.28575	-26.406
6.8	-33.305	-34.626	-33.763	-33.631	-25.028	-25.997	-33.83125	-25.5125
6.85	-34.688	-34.177	-33.355	-35.676	-24.477	-25.772	-34.474	-25.1245
6.9	-30.289	-33.377	-33.863	-37.151	-25.749	-25.543	-33.67	-25.646
6.95	-31.105	-34.679	-32.283	-36.071	-25.021	-23.518	-33.5345	-24.2695
7	-32.434	-33.737	-29.132	-33.027	-22.919	-23.993	-32.0825	-23.456
7.05	-30.100	-32.684	-30.563	-32.401	-24.807	-24.512	-31.437	-24.6595
7.1	-34.241	-31.210	-31.586	-33.127	-23.434	-25.878	-32.541	-24.656
7.15	-31.694	-32.036	-31.532	-33.322	-24.136	-22.234	-32.146	-23.185
7.2	-31.874	-30.632	-30.145	-30.857	-24.377	-25.765	-30.877	-25.071
7.25	-32.514	-29.876	-31.869	-31.030	-22.643	-23.102	-31.32225	-22.8725

7.3	-29.753	-30.655	-29.025	-32.090	-23.199	-24.838	-30.38075	-24.0185
7.35	-28.624	-29.034	-29.172	-28.955	-22.878	-25.586	-28.94625	-24.232
7.4	-27.036	-28.301	-28.008	-27.733	-22.333	-23.162	-27.7695	-22.7475
7.45	-29.064	-29.968	-29.707	-31.511	-22.644	-24.445	-30.0625	-23.5445
7.5	-28.950	-29.150	-27.203	-30.459	-21.496	-22.593	-28.9405	-22.0445
7.55	-27.270	-28.542	-27.488	-29.544	-23.315	-20.960	-28.211	-22.1375
7.6	-29.121	-25.764	-27.458	-29.850	-20.705	-23.311	-28.04825	-22.008
7.65	-30.420	-26.463	-29.269	-28.320	-24.419	-22.151	-28.618	-23.285
7.7	-27.992	-26.565	-25.993	-30.235	-23.257	-23.387	-27.69625	-23.322
7.75	-31.716	-28.392	-26.900	-26.773	-21.258	-20.217	-28.44525	-20.7375
7.8	-27.089	-26.498	-26.873	-29.341	-22.528	-24.048	-27.45025	-23.288
7.85	-27.954	-27.030	-27.739	-28.199	-22.386	-20.442	-27.7305	-21.414
7.9	-27.834	-27.780	-26.429	-27.132	-23.176	-22.647	-27.29375	-22.9115
7.95	-26.591	-28.181	-26.054	-25.418	-22.828	-22.758	-26.561	-22.793
8	-25.331	-26.462	-27.475	-25.250	-24.764	-22.586	-26.1295	-23.675
8.05	-26.181	-24.839	-26.501	-26.179	-22.341	-21.424	-25.925	-21.8825
8.1	-27.730	-26.601	-24.419	-26.250	-22.871	-21.659	-26.25	-22.265
8.15	-26.550	-26.432	-25.099	-26.885	-21.388	-21.982	-26.2415	-21.685
8.2	-28.247	-26.232	-25.562	-27.120	-22.215	-21.427	-26.79025	-21.821
8.25	-25.294	-26.514	-25.771	-24.694	-21.201	-22.017	-25.56825	-21.609
8.3	-26.743	-27.725	-26.439	-26.619	-20.859	-19.638	-26.8815	-20.2485
8.35	-26.863	-25.754	-25.006	-26.184	-21.477	-20.433	-25.95175	-20.955
8.4	-25.257	-27.765	-24.868	-25.126	-22.66	-20.305	-25.754	-21.4825
8.45	-26.117	-27.304	-23.960	-25.156	-22.678	-23.188	-25.63425	-22.933
8.5	-25.023	-27.893	-23.574	-25.548	-21.588	-19.633	-25.5095	-20.6105
8.55	-25.847	-24.634	-26.068	-26.112	-20.995	-22.951	-25.66525	-21.973
8.6	-26.140	-25.311	-24.498	-24.913	-20.763	-21.162	-25.2155	-20.9625
8.65	-26.413	-25.180	-22.997	-25.707	-18.882	-20.834	-25.07425	-19.858
8.7	-25.003	-25.457	-24.275	-24.035	-20.723	-20.413	-24.6925	-20.568
8.75	-25.574	-23.519	-22.193	-24.701	-20.537	-19.721	-23.99675	-20.129
8.8	-25.619	-25.972	-23.717	-21.861	-19.284	-21.535	-24.29225	-20.4095
8.85	-24.986	-23.302	-22.555	-24.861	-22.093	-22.238	-23.926	-22.1655
8.9	-26.364	-23.662	-22.504	-24.706	-18.672	-20.090	-24.309	-19.381
8.95	-24.605	-26.266	-25.051	-25.568	-22.133	-17.568	-25.3725	-19.8505
9	-24.430	-23.431	-24.347	-25.266	-19.904	-18.638	-24.3685	-19.271
9.05	-24.065	-24.876	-24.172	-21.113	-18.46	-17.544	-23.5565	-18.002
9.1	-23.732	-24.623	-23.842	-25.308	-19.952	-21.108	-24.37625	-20.53
9.15	-27.590	-23.315	-24.651	-24.509	-20.619	-21.148	-25.01625	-20.8835
9.2	-25.431	-22.982	-23.384	-23.128	-18.767	-17.316	-23.73125	-18.0415
9.25	-22.017	-24.819	-21.786	-23.565	-18.396	-19.002	-23.04675	-18.699
9.3	-24.941	-23.659	-24.352	-22.800	-18.664	-15.939	-23.938	-17.3015
