# Supplementary material for: Conformational Coupling between Receptor and Kinase Binding Sites through a Conserved Salt Bridge in a Signaling Complex Scaffold Protein
Source: PLoS Comput Biol. 2013 Nov 14;9(11):e1003337. doi: 10.1371/journal.pcbi.1003337 (PMC3828127; doi:10.1371/journal.pcbi.1003337)
Supplement: Table S2 — Top 5 most conserved residues in F1 and F7 classes of the CheW protein. (PDF) [file pcbi.1003337.s007.pdf]

Table S2: Top 5 most conserved residues in F1 and F7 classes of the CheW protein.

F1		F7	
Residue[1]	Identity (%)	Residue[1]	Identity (%)
Gly57	100.0	Gly57	99.8
Pro49[2]	98.7	**Arg62**	99.8
Val102	97.1	**Glu38**	99.3
Gly63	94.0	Gly63	99.1
Phe22	93.8	Gly99	99.1

[1]Residue numbers are given in reference to the *E. coli* CheW protein.

[2]Position 49 is alanine in *E. coli* CheW but it is 98.7% conserved as proline in class F1.
