# Supplementary material for: Conformational Coupling between Receptor and Kinase Binding Sites through a Conserved Salt Bridge in a Signaling Complex Scaffold Protein
Source: PLoS Comput Biol. 2013 Nov 14;9(11):e1003337. doi: 10.1371/journal.pcbi.1003337 (PMC3828127; doi:10.1371/journal.pcbi.1003337)
Supplement: Table S3 — 15N HSQC Chemical Shift assignment in 30C° for the wild-type, R62A and E38A alleles. Buffer: 30 mM Tris-HCl (pH 7.3), 30 mM NaCl, 0.2% sodium azide in 90% H2O and 10% D2O. (PDF) [file pcbi.1003337.s008.pdf]

Table S3: ^{15}N HSQC Chemical Shift assignment in 30C° for the wild-type, R62A and E38A alleles. Buffer: 30 mM Tris-HCl (pH 7.3), 30 mM NaCl, 0.2% sodium azide in 90% H_2O and 10% D_2O.

	Wild type		E38A		R62A	
Residues	H (ppm)	N (ppm)	H (ppm)	N (ppm)	H (ppm)	N (ppm)
Thr8	8.202	118.42	8.213	118.41	8.213	118.4
Lys9	8.246	124.39	-	-	8.256	124.37
Leu10	8.307	124.2	8.317	124.19	8.313	124.14
Ala11	8.309	124.81	8.321	124.82	8.32	124.8
Ser12	8.098	114.28	8.106	114.26	8.108	114.29
Glu13	8.241	123.53	8.253	123.57	8.253	123.52
Pro14		-		-		-
Ser15	8.466	116.29	8.471	116.31	8.48	116.29
Gly16	8.227	110.44	8.244	110.44	8.237	110.45
Gln17	8.374	120.55	-	-	8.378	120.56
Glu18	8.01	121.67	-	-	8.016	121.68
Phe19	9.232	120.81	9.255	120.86	9.243	120.83
Leu20	9.188	124.08	9.21	124.22	-	-
Val21	8.497	128.28	8.501	128.3	8.515	128.27
Phe22	9.344	123.81	9.32	123.76	9.35	123.8
Thr23	8.739	112.05	8.702	112.22	8.742	112.14
Leu24	8.301	124.01	8.32	123.97	8.317	124.16
Gly25	9.852	117.56	9.809	117.47	9.849	117.55
Asp26	8.718	124.75	-	-	8.738	124.83
Glu27	7.843	119.56	-	-	7.857	119.6
Glu28	7.954	118.41	7.974	118.5	7.977	118.45
Tyr29	9.006	121.93	8.996	121.89	9.011	121.91

Gly30	8.622	128.53	-	-	-	-
Ile31	9.003	118.46	9.006	118.8	9.008	118.42
Asp32	8.801	126.6	8.795	126.83	8.808	126.54
Ile33	7.951	127.64	7.994	127.77	7.95	127.62
Leu34	8.569	122.17	-	-	8.575	122.15
Lys35	7.786	115.29	7.801	115.33	7.78	115.26
Val36	7.575	120.05	7.568	120.13	7.579	120.03
Gln37	9.52	128.61	-	-	9.511	128.81
Glu38*	7.669	112.67	-	-	7.66	112.96
Ile39	8.564	122.36	-	-	8.549	122.38
Arg40	9.454	126.49	-	-	9.42	126.26
Gly41	8.243	107.49	8.47	107.66	8.337	107.37
Tyr42	8.444	121	-	-	8.42	121.31
Asp43	7.533	125.96	-	-	-	-
Gln44	8.14	117.19	-	-	8.143	117.24
Val45	7.946	123.17	-	-	-	-
Thr46	9.037	124.29	-	-	-	-
Arg47	8.616	127.83	-	-	-	-
Ile48	8.408	124.66	-	-	8.421	124.52
Ala49	8.633	129.71	-	-	8.621	129.39
Asn50	8.667	113.13	-	-	8.672	113.33
Thr51	7.475	107.32	-	-	7.507	107.72
Pro52		-		-		-
Ala53	8.076	122.81	-	-	8.088	122.84
Phe54	7.086	107.42	-	-	-	-
Ile55	7.441	123.21	-	-	-	-

Lys56	8.645	125.88	-	-	8.645	125.76
Gly57	7.153	127.42	-	-	-	-
Val58	8.614	112.56	-	-	-	-
Thr59	8.549	116.41	-	-	8.568	116.27
Asn60	8.123	124.14	-	-	-	-
Leu61	9.175	128.58	-	-	-	-
Arg62*	9.086	123.15	-	-	-	-
Gly63	8.012	130.08	-	-	-	-
Val64	7.536	122.62	-	-	-	-
Ile65	8.491	127.23	8.49	127.31	8.472	127.32
Val66	8.947	130.7	-	-	8.912	130.44
Pro67		-		-		-
Ile68	-	-	-	-	-	-
Val69	9.202	126.71	-	-	-	-
Asp70	9.765	126.48	-	-	9.69	126.36
Leu71	-	-	-	-	-	-
Arg72	8.496	117.5	8.486	117.53	8.491	117.42
Ile73	6.888	116.02	-	-	6.98	116.4
Lys74	-	-	-	-	-	-
Phe75	7.631	113.25	-	-	7.633	113.48
Ser76	7.781	112.87	-	-	7.824	112.96
Gln77	8.354	119.22	8.325	119.13	8.369	119.31
Val78	7.716	117.53	7.752	117.56	7.764	117.78
Asp79	8.148	121.64	-	-	8.159	121.68
Val80	-	-	-	-	-	-
Asp81	7.956	122.94	-	-	-	-

Tyr82	8.154	120.2	-	-	8.158	120.43
Asn83	9.14	121.24	9.065	120.88	9.109	121.15
Asp84	-	-	-	-	-	-
Asn85	8.692	116.3	8.692	116.47	8.694	116.41
Thr86	7.67	118.83	7.65	118.08	7.669	118.42
Val87	-	-	-	-	-	-
Val88	8.679	122.76	-	-	8.762	122.19
Ile89	9.005	126.16	-	-	9.02	126.17
Val90	9.131	128.44	-	-	-	-
Leu91	9.511	129.28	9.48	129.15	9.491	129.33
Asn92	8.672	118.69	8.671	118.56	8.691	118.74
Leu93	-	-	-	-	-	-
Gly94	8.661	112.77	-	-	8.656	112.6
Gln95	8.555	121.45	8.576	121.4	8.557	121.5
Arg96	7.472	117.09	7.56	117.4	7.446	116.9
Val97	7.991	122.72	-	-	8.018	122.57
Val98	8.516	122.88	-	-	8.535	122.88
Gly99	9.141	113.01	9.099	112.77	9.115	112.83
Ile100	8.996	116.78	8.981	116.86	-	-
Val101	8.778	123.82	-	-	8.706	123.69
Val102	8.293	116.55	-	-	-	-
Asp103	7.968	117.08	7.976	117.24	7.963	117.13
Gly104	7.424	129.57	-	-	7.418	104.39
Val105	8.591	117.19	-	-	8.585	117.18
Ser106	8.875	120.54	8.895	120.56	8.884	120.5
Asp107	8.108	120.2	-	-	8.107	120.17

Val108	8.074	117.92	8.103	117.95	8.088	117.94
Leu109	8.973	126.74	8.982	126.75	8.979	126.7
Ser110	8.376	116.72	8.397	116.81	8.387	116.71
Leu111	8.772	124.89	8.771	124.83	8.776	124.86
Thr112	8.735	113.54	8.747	113.55	8.744	113.55
Ala113	8.664	121.96	8.675	121.98	8.675	121.97
Glu114	8.323	114.23	8.337	114.27	8.337	114.25
Gln115	7.793	117.97	7.798	117.98	7.804	117.96
Ile116	7.254	120.14	7.269	120.17	7.263	120.13
Arg117	9.171	128.39	-	-	-	-
Pro118		-		-		-
Ala119	8.362	122.29	-	-	-	-
Pro120		-		-		-
Glu121	8.591	119.51	-	-	8.606	119.51
Phe122	7.183	116.35	7.253	116.43	7.18	116.34
Ala123	8.177	123.72	8.153	123.82	8.192	123.73
Val124	8.27	116.64	-	-	-	-
Thr125	8.553	115.92	8.557	115.74	8.578	115.97
Leu126	7.19	120.45	7.227	120.38	7.188	120.24
Ser127	7.305	113.88	7.445	114	7.318	113.84
Thr128	8.143	116.75	-	-	8.151	116.83
Glu129	8.716	120.86	8.693	120.91	-	-
Tyr130	7.578	115.3	7.567	115.21	7.593	115.36
Leu131	-	-	-	-	-	-
Thr132	8.928	115.15	8.943	115.4	8.939	115.11
Gly133	7.445	106.88	7.47	106.9	7.452	106.85

Leu134	-	-	-	-	-	-
Gly135	9.778	112.07	9.785	112	9.802	112.12
Ala136	8.678	128.37	8.699	128.37	8.69	128.41
Leu137	8.073	125.51	8.089	125.48	8.077	125.53
Gly138	8.604	114.76	8.612	114.66	8.614	114.76
Asp139	8.62	125.44	-	-	8.632	125.48
Arg140	7.771	121.45	-	-	7.781	121.45
Met141	8.409	124.27	8.415	124.1	8.423	124.26
Leu142	9.034	124.53	-	-	9.043	124.53
Ile143	8.41	126.71	8.444	126.76	8.444	126.75
Leu144	8.322	128.39	8.336	128.34	8.336	128.4
Val145	8.319	121.72	-	-	-	-
Asn146	9.187	124.89	-	-	9.207	124.98
Ile147	-	-	-	-	-	-
Glu148	8.314	122.83	-	-	-	-
Lys149	7.369	117.12	7.412	116.69	7.369	117.22
Leu150	7.51	118.61	7.439	118.76	7.522	118.63
Leu151	7.695	115.67	7.719	115.5	7.724	115.72
Asn152	-	-	-	-	-	-
Ser153	-	-	-	-	-	-
Glu154	8.594	123.9	-	-	-	-
Glu155	8.616	119.8	-	-	8.603	119.78
Met156	8.139	116.99	-	-	8.152	116.98
Ala157	7.672	120.94	-	-	7.689	121.05
Leu158	-	-	-	-	-	-
Leu159	7.88	120.18	-	-	7.929	120.44

Asp160	8.173	119.83	-	-	8.188	119.89
Ser161	7.986	115.39	7.979	115.33	8.017	115.52
Ala162	8.016	124.76	-	-	8.045	124.83
Ala163	7.972	119.94	-	-	7.984	119.94
Ser164	7.859	113.45	-	-	7.878	113.5
Glu165	8.086	122.14	-	-	8.106	122.18
Val166	7.884	119.71	-	-	7.895	119.74
Ala167	-	-	-	-	-	-

* mutated residues
