# Supplementary material for: Conformational Coupling between Receptor and Kinase Binding Sites through a Conserved Salt Bridge in a Signaling Complex Scaffold Protein
Source: PLoS Comput Biol. 2013 Nov 14;9(11):e1003337. doi: 10.1371/journal.pcbi.1003337 (PMC3828127; doi:10.1371/journal.pcbi.1003337)
Supplement: Table S4 — Differences phenomenological transverse relaxation rate constant ΔRex = Rex(20 ms)−Rex(1 ms) and ΔΔRex the differences between the ΔRex in R62A and wild-type. Residues with large ΔΔRex in bold are likely to be involved in motions in the microsecond-to-millisecond time scale. (PDF) [file pcbi.1003337.s009.pdf]

Table S4: Differences phenomenological transverse relaxation rate constant ΔR_{ex} = R_{ex}(20ms) –R_{ex}(1ms) and $\Delta\Delta R_{ex}$ the differences between the ΔR_{ex} in R62A and Wild type. Residues with large $\Delta\Delta R_{ex}$ are likely to be involved in motions in the microsecond-to-millisecond time scale (bold).

	ΔRex (s^{-1})		$\Delta\Delta R_{ex}$ (s^{-1})
Residues	Wild type	R62A	ΔR_{ex}(R62A)- ΔR_{ex}(WT)
Thr23		2.83	2.83
Asp26	1.9	2.19	0.29
Glu27		1.44	1.44
Tyr29	2.59	2.92	0.33
Ile31		3.32	3.32
Glu38	1.56	4.41	2.85
Ile39	1.57	5.78	4.21
Arg40		4.3	4.3
Gly41		4.91	4.91
Tyr42	2.12	3.03	0.91
Val45	12.87	11.75	-1.12
Ile48	2.68	2.9	0.22
Ala49	6.27		-6.27
Asn50	6.19		-6.19
Ile55	6.09		-6.09
Thr59	9.56		-9.56
Gly63	8.15		-8.15
**Ile65**	**2.66**	**11.09**	**8.43**
Val66	9.09	16.91	7.82
Arg72	3.79	6.45	2.66
Ile73	3.21	4.44	1.23
**Phe75**	**8.51**	**13.67**	**5.16**
**Ser76**	**3.26**	**12.08**	**8.82**
**Gln77**	**2.57**	**8.95**	**6.38**
Val78	7.09	8.84	1.75

Asp79	2.32	3.09	0.77
Asp81	8.83		-8.83
Asn83	1.83	4.69	2.86
Asn85	1.19	2.08	0.89
Thr86	5.33	16.87	11.54
Val88	5.89		-5.89
Gly94	6.08		-6.08
Gln95	5.75	7.74	1.99
Val97	4.55	6.01	1.46
Val98	2.78	3.91	1.13
Gly99	2.78	7.84	5.06
Ile100	9.31		-9.31
Ser106	1.53	2.55	1.02
Asp107	1.54	0.7	-0.84
Val108	0.67	0.56	-0.11
Leu111	4.23	3.55	-0.68
Glu114	1.31	1.39	0.08
Ala119	1.74		-1.74
Glu121	5.14	5.47	0.33
Phe122	3.38	4.4	1.02
Ala123	3.83	3.58	-0.25
Thr125	6.68		-6.68
Glu129	4.59	5.04	0.45
Tyr130	10.35		-10.35
Ala136	6.09	6.68	0.59
Ala136		4.92	4.92
Arg140	1.7	1.73	0.03
Met141		0.59	0.59
Leu142	2.13		-2.13

Val145	4.94		-4.94
Asn146	5.34	6.34	1
Lys149	10.54	14.8	4.26
Leu150	8.1	6.96	-1.14
Leu151		3.51	3.51
Glu155	9.65	6.82	-2.83
Ala157	9.21		-9.21
**Leu159**	**5.35**	**13.13**	**7.78**
Asp160	7.65	12.46	4.81
Ser161	9.02	11.08	2.06
Ala162	2.89	5.62	2.73
**Ser164**	**6.8**	**15.94**	**9.14**
Glu165	1.07	3.29	2.22
Val166	6.17	13.27	7.1
