# Supplementary material for: Conformational Coupling between Receptor and Kinase Binding Sites through a Conserved Salt Bridge in a Signaling Complex Scaffold Protein
Source: PLoS Comput Biol. 2013 Nov 14;9(11):e1003337. doi: 10.1371/journal.pcbi.1003337 (PMC3828127; doi:10.1371/journal.pcbi.1003337)
Supplement: Table S5 — Root mean square deviation per residue of all residues for each simulation. Frames were aligned using backbone atoms of the residues Ile55 to Val68, which is proposed to be the CheA binding site. We use T-test to find RMSD per residue values significantly different from wild-type and R62A simulations. Values with p-value<0.00002 are shown in bold. (PDF) [file pcbi.1003337.s010.pdf]

Table S5: Root mean square deviation per residue of all residues for each simulation. Frames were aligned using backbone atoms of the residues Ile55 to Val68, which is proposed to be the CheA binding site. We use T-test to find RMSD per residue values significantly different from wild-type and R62A simulations. Values with p-value < 0.00002 are shown in bold.

	RMSD per residue (Å)																				Statistics				
	Simulations with wild-type allele										Simulations with R62A mutant										Wild-type		R62A		T-Test
Residue	1	2	3	4	5	6	7	8	9	10	1	2	3	4	5	6	7	8	9	10	Mean	STDEV	Mean	STDEV	p-value
1	10.30	10.10	6.54	19.19	8.39	18.13	8.70	15.49	15.82	10.77	11.89	9.97	6.84	12.95	7.78	14.11	17.25	7.18	10.44	22.41	12.34	4.43	12.08	4.89	0.901706582
2	10.63	11.30	8.02	20.80	9.16	18.00	9.23	17.21	15.08	6.11	10.92	10.18	6.80	13.42	8.31	15.86	17.02	8.54	11.84	24.37	12.55	4.89	12.73	5.24	0.940027556
3	8.30	9.90	6.98	19.29	8.30	18.16	8.25	14.90	13.90	5.65	9.25	9.44	6.72	11.45	8.77	14.40	16.84	8.71	13.02	23.89	11.36	4.84	12.25	5.10	0.695042617
4	6.27	7.96	5.47	16.05	6.61	18.22	6.64	14.79	13.03	4.33	8.30	8.77	6.61	11.08	8.46	12.16	16.61	8.72	15.54	21.22	9.94	5.05	11.75	4.66	0.41561364
5	6.58	8.13	5.99	15.71	8.02	19.20	7.56	10.69	13.60	5.28	10.16	9.39	7.88	9.05	11.28	12.21	18.19	9.83	16.32	22.61	10.08	4.64	12.69	4.78	0.230504856
6	5.84	7.55	5.35	13.95	8.77	17.73	7.11	11.06	13.09	5.07	12.66	11.36	8.09	7.08	11.13	10.58	17.48	10.37	14.53	23.05	9.55	4.26	12.63	4.72	0.142766574
7	7.37	6.56	5.20	11.46	8.92	16.54	5.60	10.83	13.55	6.44	11.97	10.44	8.14	7.33	11.62	10.37	16.78	11.57	16.37	22.67	9.25	3.76	12.73	4.63	0.081644312
8	7.29	5.17	4.53	11.31	8.86	15.15	6.28	8.95	12.02	6.14	10.54	9.76	7.88	7.08	10.42	8.59	14.23	11.01	13.91	20.07	8.57	3.39	11.35	3.84	0.103856648
9	10.02	5.57	6.36	10.04	11.99	14.51	8.84	11.39	13.43	8.80	12.18	12.32	9.80	7.99	12.86	9.85	15.95	12.55	14.01	21.43	10.09	2.86	12.89	3.76	0.077342457
10	8.15	5.38	4.66	8.76	9.95	13.47	6.97	8.15	11.20	6.85	9.24	9.78	9.11	9.24	10.13	7.73	12.17	10.44	10.97	17.72	8.35	2.66	10.65	2.76	0.073988382
11	7.42	5.34	4.59	7.93	6.99	12.79	5.82	7.01	9.04	6.10	8.41	9.85	7.49	6.84	9.98	7.03	11.12	11.20	9.55	17.03	7.30	2.32	9.85	2.98	0.046827349
12	6.33	4.84	5.06	6.84	6.87	10.16	5.00	5.69	6.99	4.79	6.81	8.27	6.33	6.36	8.18	6.53	9.24	9.39	7.95	15.18	6.26	1.63	8.42	2.63	0.040116133
13	5.90	5.49	5.22	6.42	6.66	8.98	6.48	4.99	5.76	4.83	5.82	6.56	6.36	6.12	7.28	6.20	7.87	8.89	7.52	12.70	6.07	1.20	7.53	2.05	0.067695447
14	5.15	3.90	4.53	5.30	4.62	7.46	5.67	3.76	4.70	3.83	5.06	5.41	5.00	5.93	6.66	4.89	6.39	7.46	6.29	9.77	4.89	1.11	6.29	1.48	0.028367982
15	3.89	2.83	3.50	4.03	3.53	4.24	4.52	2.80	3.63	2.87	4.07	4.23	4.02	4.06	5.30	3.60	4.96	5.68	5.03	6.38	3.58	0.61	4.73	0.88	0.003240917
16	3.21	2.35	2.98	3.04	2.97	2.75	3.92	2.31	2.96	2.36	3.88	3.36	3.49	3.25	4.53	3.01	3.97	4.61	4.00	3.60	2.88	0.49	3.77	0.53	0.001044055
17	3.02	2.36	2.84	2.76	2.65	2.47	3.23	2.23	2.84	2.34	3.91	3.04	3.20	3.12	4.14	2.73	3.76	4.25	3.48	2.86	2.67	0.32	3.45	0.54	0.00104658
**18**	**2.64**	**1.99**	**2.27**	**2.24**	**2.38**	**2.09**	**2.75**	**1.98**	**2.19**	**2.28**	**3.79**	**3.08**	**2.85**	**3.61**	**3.90**	**2.50**	**3.19**	**3.99**	**3.25**	**2.56**	**2.28**	**0.25**	**3.27**	**0.54**	**5.45659E-05**
19	2.32	1.65	2.01	1.97	1.96	1.70	2.47	1.68	2.01	1.67	3.03	2.44	2.51	2.44	3.34	2.04	2.68	3.52	2.61	2.19	1.94	0.28	2.68	0.48	0.000556736
20	1.95	1.36	1.63	1.54	1.74	1.50	1.98	1.34	1.51	1.41	2.41	2.29	2.01	2.79	2.83	1.69	2.24	3.17	2.10	1.84	1.60	0.23	2.34	0.47	0.000286339
**21**	**1.91**	**1.43**	**1.72**	**1.56**	**1.66**	**1.47**	**1.76**	**1.44**	**1.61**	**1.46**	**2.50**	**2.48**	**2.09**	**3.32**	**3.07**	**1.81**	**2.39**	**3.40**	**2.11**	**2.11**	**1.60**	**0.16**	**2.53**	**0.55**	**7.78549E-05**
22	1.74	1.29	1.59	1.43	1.39	1.30	1.37	1.28	1.40	1.24	1.90	2.11	1.69	2.71	2.48	1.54	1.96	2.75	1.73	1.76	1.40	0.15	2.06	0.44	0.000284271
23	2.12	1.80	1.91	1.66	1.66	1.60	1.60	1.62	1.82	1.50	2.24	2.64	1.92	3.74	2.69	1.91	2.41	3.22	2.04	2.08	1.73	0.19	2.49	0.60	0.001252564
24	1.96	1.53	1.99	1.66	1.72	1.71	1.60	1.64	1.70	1.56	1.97	2.70	1.76	3.84	2.54	1.83	2.27	3.01	2.05	2.06	1.71	0.15	2.40	0.64	0.003780757
25	2.45	1.78	2.46	2.24	1.75	2.10	2.23	2.35	2.06	1.65	2.48	3.24	2.10	4.83	3.20	2.17	2.93	3.41	2.91	2.18	2.11	0.30	2.95	0.82	0.006846461
26	3.13	2.35	2.96	3.07	2.25	2.70	3.45	3.08	2.82	2.14	2.98	3.49	2.54	5.83	4.22	2.74	3.50	3.91	3.73	2.64	2.79	0.43	3.56	0.98	0.036516081
27	3.03	2.31	2.68	2.87	2.45	2.44	3.29	2.93	2.61	2.17	2.61	3.06	2.36	4.79	3.51	2.55	3.23	3.60	3.36	2.76	2.68	0.35	3.18	0.71	0.057988607
28	2.53	1.86	2.32	2.13	2.08	2.03	1.95	1.99	2.15	1.78	2.59	2.83	2.39	4.45	3.35	2.18	2.89	3.55	2.39	2.70	2.08	0.22	2.93	0.68	0.001494312
29	2.07	1.55	1.84	1.76	1.70	1.62	1.56	1.75	1.78	1.43	2.08	2.33	1.89	3.44	2.75	1.85	2.22	3.04	2.10	2.11	1.70	0.18	2.38	0.53	0.001140855
30	2.09	1.47	1.73	1.71	1.63	1.48	1.79	1.46	1.73	1.38	2.33	2.06	2.04	2.20	2.82	1.75	2.14	2.98	1.96	1.88	1.65	0.21	2.22	0.40	0.000845879
31	1.95	1.33	1.50	1.87	1.45	1.31	1.71	1.27	1.54	1.23	2.17	1.86	1.86	1.84	2.53	1.56	1.96	2.73	1.88	1.59	1.52	0.25	2.00	0.38	0.00365465
32	2.16	1.78	1.98	1.93	1.91	1.84	2.25	1.87	1.92	1.70	2.76	2.43	2.29	2.28	3.13	1.96	2.49	3.18	2.48	1.88	1.93	0.17	2.49	0.43	0.001373336
**33**	**2.05**	**1.50**	**1.60**	**1.69**	**1.67**	**1.53**	**2.23**	**1.46**	**1.62**	**1.42**	**2.88**	**2.44**	**2.25**	**3.11**	**3.01**	**1.87**	**2.38**	**3.18**	**2.50**	**1.86**	**1.68**	**0.26**	**2.55**	**0.48**	**9.12068E-05**
**34**	**2.38**	**1.96**	**2.00**	**2.03**	**1.91**	**2.01**	**2.56**	**1.92**	**2.05**	**1.80**	**3.34**	**2.79**	**2.60**	**3.46**	**3.27**	**2.16**	**2.68**	**3.33**	**2.93**	**2.13**	**2.06**	**0.23**	**2.87**	**0.48**	**0.000151678**
35	2.07	1.54	1.57	1.59	1.60	1.57	2.07	1.50	1.69	1.38	2.56	2.24	2.10	2.65	2.63	1.70	2.27	2.76	2.40	1.63	1.66	0.23	2.29	0.39	0.000322403
**36**	**1.56**	**1.09**	**1.12**	**1.15**	**1.12**	**1.05**	**1.72**	**0.99**	**1.15**	**0.99**	**2.13**	**2.04**	**1.72**	**2.67**	**2.35**	**1.36**	**1.88**	**2.51**	**1.91**	**1.41**	**1.19**	**0.24**	**2.00**	**0.43**	**7.45504E-05**
**37**	**1.67**	**1.06**	**1.01**	**1.31**	**1.01**	**0.97**	**1.78**	**0.98**	**1.09**	**1.00**	**2.17**	**2.42**	**1.87**	**3.65**	**2.31**	**1.56**	**2.05**	**2.36**	**2.25**	**1.99**	**1.19**	**0.30**	**2.26**	**0.55**	**3.83027E-05**
**38**	**1.35**	**0.96**	**1.06**	**1.06**	**1.06**	**0.94**	**1.92**	**0.90**	**1.07**	**0.90**	**2.29**	**2.50**	**1.77**	**3.58**	**2.23**	**1.41**	**1.84**	**2.45**	**2.15**	**1.66**	**1.12**	**0.31**	**2.19**	**0.60**	**0.00010252**
**39**	**1.28**	**1.01**	**1.13**	**1.00**	**1.09**	**1.05**	**1.62**	**1.03**	**1.01**	**0.99**	**2.08**	**2.49**	**1.67**	**3.62**	**2.35**	**1.35**	**1.95**	**2.59**	**1.93**	**1.65**	**1.12**	**0.20**	**2.17**	**0.64**	**0.000109705**
40	1.51	1.14	1.25	1.16	1.11	1.13	2.15	1.91	1.17	1.09	2.21	2.69	1.70	3.87	2.71	1.48	1.96	2.89	2.29	1.57	1.36	0.38	2.34	0.73	0.001448727
41	1.15	0.93	1.27	0.98	0.95	0.96	1.50	0.92	0.96	0.92	1.98	2.47	1.51	3.86	1.91	1.24	1.90	2.21	1.78	1.39	1.05	0.20	2.02	0.74	0.000865042
42	1.07	0.92	1.43	0.95	0.94	1.00	1.21	0.96	1.00	0.94	1.84	2.21	1.64	3.49	1.89	1.16	2.05	1.70	1.49	1.26	1.04	0.16	1.87	0.66	0.001056716
43	1.66	1.45	1.84	1.52	1.53	1.50	2.33	1.54	1.51	1.54	2.26	2.95	2.04	4.04	2.47	1.62	2.37	2.50	2.03	1.83	1.64	0.26	2.41	0.68	0.003868304
44	1.74	1.51	1.75	1.70	1.53	1.60	2.01	1.59	1.82	1.52	1.82	2.87	1.85	4.11	2.58	1.81	1.93	2.81	1.82	1.73	1.68	0.16	2.33	0.77	0.016520131
45	1.05	0.77	0.93	1.01	0.98	0.85	0.98	0.86	1.09	0.79	1.20	1.42	0.94	1.76	1.42	0.90	0.97	1.55	0.93	0.79	0.93	0.11	1.19	0.33	0.031613679
46	1.34	1.06	1.26	1.20	1.10	1.06	1.25	1.07	1.51	1.10	1.35	1.58	1.14	1.41	1.58	1.05	1.03	1.47	1.01	0.94	1.20	0.15	1.26	0.25	0.51569051
47	3.16	3.15	2.50	3.16	2.79	3.11	3.61	3.31	3.36	2.77	3.17	4.44	3.72	3.30	3.33	3.32	2.83	3.79	3.02	2.42	3.09	0.32	3.33	0.56	0.251765131
48	3.24	3.05	3.66	3.29	2.39	3.14	3.36	3.59	4.11	2.60	3.05	3.95	3.08	3.28	3.53	3.26	2.50	2.87	2.57	2.64	3.24	0.50	3.07	0.45	0.444027759
49	3.70	3.58	4.11	4.19	2.59	4.20	4.31	3.93	3.29	3.07	4.11	3.67	4.15	3.71	4.29	3.97	3.15	3.81	3.36	3.21	3.70	0.57	3.74	0.40	0.82977404
50	3.60	3.81	5.14	3.68	4.12	5.42	3.44	6.70	3.60	4.00	7.20	4.42	3.60	3.61	4.98	4.87	5.05	6.04	3.38	5.11	4.35	1.06	4.83	1.18	0.355531289
51	3.24	3.01	3.88	2.76	3.31	4.16	2.92	5.42	3.27	3.06	4.75	3.52	3.09	3.33	4.37	4.15	5.50	6.21	3.50	6.09	3.50	0.80	4.45	1.15	0.04635002
52	2.43	2.63	4.37	2.35	2.98	4.01	2.58	4.80	2.65	3.13	5.56	3.34	3.31	2.71	4.22	4.15	5.76	6.05	4.00	6.38	3.19	0.88	4.55	1.29	0.013526265
53	1.40	1.41	3.40	1.30	1.51	1.99	1.45	3.11	1.33	2.38	2.77	2.13	2.11	1.38	2.65	2.22	2.87	2.90	2.41	3.41	1.93	0.78	2.49	0.56	0.082480273
54	1.99	1.97	2.44	1.99	2.03	2.65	2.89	3.73	2.02	3.72	2.34	4.80	3.61	1.98	2.72	2.11	2.55	2.07	2.66	3.11	2.54	0.70	2.80	0.87	0.48279942
55	0.63	0.52	1.52	0.52	0.57	0.67	0.57	0.58	0.65	0.63	1.29	1.59	0.99	0.99	1.15	0.95	1.29	1.15	0.92	1.44	0.69	0.30	1.18	0.22	0.00059089

56	0.63	0.70	0.88	0.69	0.73	0.90	0.71	0.85	0.75	0.73	1.04	1.33	0.82	1.52	0.84	0.74	1.10	1.03	0.86	0.99	0.76	0.09	1.03	0.24	0.004078141
57	0.34	0.34	0.41	0.34	0.36	0.34	0.37	0.36	0.38	0.33	0.54	0.50	0.44	0.47	0.50	0.38	0.38	0.51	0.37	0.43	0.36	0.03	0.45	0.06	0.000256802
58	0.73	0.66	0.71	0.89	0.70	0.67	0.81	0.79	0.69	0.83	0.78	0.87	0.80	0.90	0.99	0.77	0.73	0.87	0.74	0.93	0.75	0.08	0.84	0.09	0.020865585
59	0.48	0.41	0.46	0.49	0.42	0.42	0.43	0.42	0.50	0.40	0.55	0.61	0.51	0.64	0.86	0.51	0.50	0.90	0.50	0.52	0.44	0.04	0.61	0.15	0.003028286
60	1.07	1.07	1.11	1.03	1.08	1.06	1.15	1.16	1.08	1.05	1.15	1.10	1.11	1.15	1.59	1.17	1.19	1.54	1.12	1.14	1.09	0.04	1.23	0.18	0.030002121
61	0.79	0.72	0.75	0.84	0.77	0.75	1.12	0.62	0.84	0.68	1.17	1.20	0.86	1.12	2.05	0.92	1.04	2.76	1.17	0.97	0.79	0.14	1.33	0.60	0.013440386
62	0.93	0.59	0.67	0.70	0.62	0.64	1.39	0.65	0.69	0.58	0.78	0.92	0.74	0.76	0.95	0.80	0.81	1.43	0.78	0.84	0.75	0.25	0.88	0.20	0.202013692
**63**	**0.52**	**0.40**	**0.50**	**0.46**	**0.42**	**0.42**	**0.49**	**0.45**	**0.47**	**0.39**	**0.60**	**0.72**	**0.59**	**0.64**	**0.66**	**0.55**	**0.64**	**0.84**	**0.60**	**0.64**	**0.45**	**0.04**	**0.65**	**0.08**	**2.94062E-06**
64	0.96	0.50	0.69	0.63	0.57	0.56	0.64	0.56	0.61	0.54	0.70	0.72	0.66	0.81	0.80	0.64	0.75	1.13	0.61	0.83	0.62	0.13	0.77	0.15	0.035104777
65	1.10	1.01	1.09	1.06	1.02	1.03	1.10	1.01	1.02	1.08	1.12	1.07	1.03	1.14	1.09	1.00	1.03	1.13	1.05	1.05	1.05	0.04	1.07	0.05	0.335264891
66	0.42	0.36	0.41	0.59	0.38	0.38	0.39	0.36	0.41	0.38	0.45	0.61	0.47	0.56	0.66	0.41	0.45	0.77	0.52	0.48	0.41	0.07	0.54	0.11	0.004731736
67	0.48	0.42	0.46	0.44	0.44	0.44	0.45	0.41	0.45	0.40	0.47	0.54	0.50	0.48	0.60	0.46	0.50	0.67	0.51	0.47	0.44	0.02	0.52	0.07	0.002215982
68	0.59	0.65	0.64	0.52	0.53	0.55	0.49	0.53	0.61	0.55	0.78	0.72	0.59	0.81	0.86	0.67	0.64	0.75	0.61	0.53	0.56	0.05	0.70	0.10	0.002391538
69	0.81	0.60	0.69	0.65	0.62	0.75	0.62	0.67	0.80	0.61	0.82	0.95	0.97	1.32	1.09	0.72	0.83	1.10	0.85	0.66	0.68	0.08	0.93	0.20	0.001651878
70	1.07	1.00	1.32	0.98	1.09	1.25	0.94	1.00	1.10	1.01	1.49	1.68	1.20	2.56	1.29	1.07	1.50	1.51	1.24	0.98	1.08	0.12	1.45	0.45	0.020036786
71	1.45	0.94	1.29	1.02	1.04	1.28	0.95	1.07	1.13	0.87	1.34	1.67	1.35	2.50	1.61	1.17	1.54	1.85	1.32	1.32	1.10	0.18	1.57	0.39	0.003035597
72	1.71	1.26	1.96	1.34	1.21	1.36	1.36	1.90	1.50	1.13	2.87	2.74	2.27	4.91	1.96	1.66	2.72	2.56	1.85	1.80	1.47	0.29	2.54	0.94	0.003167876
73	2.02	1.53	1.87	1.48	1.54	1.56	1.65	1.70	1.55	1.69	2.88	2.76	2.21	4.98	2.14	1.92	2.79	2.55	1.99	2.07	1.66	0.17	2.63	0.90	0.003501288
74	1.94	1.69	1.76	1.64	1.79	1.72	1.77	1.75	1.71	1.44	2.47	2.60	2.09	3.94	2.32	1.90	2.29	2.50	2.03	2.38	1.72	0.13	2.45	0.57	0.000852274
75	2.08	1.55	1.84	1.75	1.66	1.66	1.63	1.79	1.78	1.38	2.01	2.24	1.96	3.37	2.41	1.76	2.19	2.46	2.09	2.27	1.71	0.19	2.28	0.44	0.001458127
76	2.24	1.52	1.99	1.65	1.63	1.61	1.62	1.79	1.78	1.49	2.79	3.17	2.34	5.63	2.37	1.94	2.72	2.74	2.25	2.55	1.73	0.23	2.85	1.04	0.00371323
77	2.59	1.91	2.33	2.05	1.69	1.94	2.01	2.37	2.06	1.58	2.61	3.14	2.25	5.27	2.71	2.20	3.31	3.01	2.59	2.43	2.05	0.31	2.95	0.89	0.007572988
78	2.40	1.87	3.51	1.88	1.57	1.83	1.81	2.25	2.03	1.52	3.50	3.78	2.91	7.16	2.68	2.38	4.03	3.97	2.46	2.97	2.07	0.58	3.59	1.40	0.005192976
79	2.43	1.86	4.70	1.96	1.76	1.91	1.72	2.07	2.12	1.67	3.73	4.19	2.95	7.49	2.94	2.34	4.85	4.60	2.47	3.27	2.22	0.90	3.88	1.53	0.008246641
80	2.00	1.29	3.46	1.42	1.27	1.44	1.26	1.43	1.51	1.21	3.39	2.99	2.49	5.37	2.39	1.76	4.04	3.48	1.91	2.09	1.63	0.68	2.99	1.12	0.004124803
81	2.20	1.43	3.24	1.54	1.46	1.56	1.67	1.54	1.64	1.44	3.04	3.11	2.56	5.50	2.39	1.81	4.14	3.52	2.13	1.89	1.77	0.56	3.01	1.15	0.006661452
82	2.20	1.98	3.18	1.83	1.69	2.01	2.24	2.65	2.06	1.67	3.51	3.37	2.94	5.24	2.75	2.18	3.19	2.92	2.56	2.45	2.15	0.46	3.11	0.85	0.005866178
83	2.08	1.65	2.76	1.61	1.62	1.79	1.99	1.74	1.79	1.66	2.93	2.91	2.70	4.07	2.01	1.82	2.62	2.69	2.32	1.66	1.87	0.35	2.57	0.69	0.010181951
84	2.10	1.49	3.18	1.56	1.50	1.69	2.01	1.62	1.68	1.51	2.81	3.30	2.67	5.28	2.18	1.97	3.53	3.95	2.57	1.84	1.84	0.52	3.01	1.05	0.005244492
85	1.35	1.05	1.87	1.15	1.09	1.23	1.44	1.15	1.24	1.10	2.12	2.57	1.84	4.20	1.73	1.42	2.60	2.88	1.76	1.42	1.27	0.24	2.25	0.85	0.002438702
86	1.62	1.30	1.82	1.48	1.47	1.42	1.89	1.29	1.44	1.34	2.47	3.06	2.07	4.64	2.33	1.71	2.63	2.90	2.19	1.71	1.51	0.21	2.57	0.86	0.001275051
87	1.34	1.11	1.47	1.15	1.27	1.30	1.59	1.15	1.27	1.23	1.89	2.49	1.58	3.73	2.13	1.35	1.93	2.49	1.82	1.54	1.29	0.15	2.09	0.69	0.00195358
88	1.00	0.89	1.09	0.80	0.87	1.04	1.23	0.80	0.91	0.90	1.43	2.00	1.24	2.68	1.72	1.07	1.46	1.88	1.44	1.09	0.95	0.14	1.60	0.49	0.00076913
**89**	**1.07**	**0.81**	**0.96**	**0.82**	**0.89**	**0.81**	**1.13**	**0.77**	**0.86**	**0.80**	**1.41**	**1.69**	**1.22**	**2.15**	**1.80**	**1.06**	**1.43**	**2.06**	**1.34**	**1.10**	**0.89**	**0.12**	**1.53**	**0.38**	**0.000101115**
**90**	**0.90**	**0.69**	**0.75**	**0.72**	**0.73**	**0.70**	**1.15**	**0.64**	**0.72**	**0.69**	**1.30**	**1.61**	**1.17**	**2.06**	**1.61**	**0.94**	**1.30**	**1.80**	**1.29**	**1.11**	**0.77**	**0.15**	**1.42**	**0.34**	**3.43682E-05**
91	1.45	0.95	0.99	1.10	1.03	0.99	1.37	1.12	1.09	0.90	1.55	1.47	1.36	1.73	1.80	1.12	1.47	1.95	1.50	1.23	1.10	0.18	1.52	0.26	0.000513237
92	2.10	1.30	1.31	1.53	1.25	1.33	2.00	1.28	1.35	1.31	2.06	2.31	1.90	3.54	2.10	1.57	2.15	2.31	1.77	1.79	1.48	0.31	2.15	0.54	0.003278653
93	2.85	1.72	1.78	1.86	1.58	1.77	1.91	1.62	1.73	1.85	2.29	2.77	2.15	3.43	2.30	1.81	2.32	2.51	2.01	1.84	1.87	0.36	2.34	0.48	0.022118751
94	4.11	2.83	2.28	2.10	1.86	2.36	2.68	2.30	2.55	2.25	2.84	3.35	3.19	5.87	2.52	2.32	3.72	3.15	2.49	1.79	2.53	0.62	3.12	1.12	0.160170934
95	3.45	2.39	3.33	3.30	3.12	2.93	2.63	2.62	2.69	3.22	2.84	3.96	2.99	5.50	2.85	2.58	2.98	3.36	3.31	2.48	2.97	0.37	3.28	0.89	0.314986351
96	2.15	1.55	2.00	1.64	1.67	2.06	1.88	2.34	1.80	1.91	1.92	2.65	2.16	3.72	2.02	1.67	2.12	2.26	2.12	1.76	1.90	0.24	2.24	0.59	0.108222068
97	1.26	1.02	1.13	1.23	1.10	1.16	1.20	1.08	1.16	1.12	1.39	1.57	1.36	2.52	1.59	1.17	1.35	1.71	1.56	1.45	1.15	0.07	1.57	0.37	0.002226295
**98**	**1.17**	**1.03**	**0.88**	**0.97**	**0.98**	**1.00**	**1.08**	**0.98**	**0.95**	**0.94**	**1.20**	**1.26**	**1.17**	**1.52**	**1.40**	**1.02**	**1.19**	**1.43**	**1.23**	**1.27**	**1.00**	**0.08**	**1.27**	**0.14**	**6.96149E-05**
99	0.70	0.54	0.61	0.51	0.57	0.54	0.66	0.52	0.53	0.51	0.71	0.88	0.77	0.83	1.09	0.63	0.73	1.12	0.81	0.66	0.57	0.07	0.82	0.17	0.000291115
100	0.95	0.72	0.95	0.71	0.77	0.72	0.82	0.70	0.75	0.68	1.04	1.23	0.96	1.48	1.43	0.85	1.11	1.58	0.98	0.86	0.78	0.10	1.15	0.27	0.000559097
101	1.19	1.04	1.45	1.05	1.06	1.08	1.08	1.08	1.12	1.02	1.41	1.81	1.26	2.56	1.57	1.19	1.54	1.78	1.29	1.26	1.12	0.13	1.57	0.41	0.003921256
102	1.30	0.97	1.38	1.01	1.04	1.02	1.13	1.03	1.07	0.96	1.53	2.03	1.31	3.09	1.94	1.21	1.72	2.29	1.45	1.44	1.09	0.14	1.80	0.57	0.001183504
103	1.88	1.42	1.95	1.52	1.50	1.51	1.65	1.54	1.57	1.43	2.42	2.91	1.91	4.50	2.48	1.72	2.51	3.16	2.08	2.08	1.60	0.18	2.58	0.81	0.001454907
104	1.67	1.26	1.65	1.36	1.35	1.30	1.56	1.35	1.37	1.25	2.08	2.66	1.69	3.76	2.51	1.56	2.22	3.01	1.93	1.94	1.41	0.16	2.33	0.67	0.000483164
105	1.85	1.54	1.80	1.59	1.61	1.54	1.92	1.55	1.61	1.55	2.44	2.86	2.05	3.95	2.87	1.80	2.48	3.32	2.22	2.20	1.66	0.14	2.62	0.65	0.000223863
106	1.99	1.68	2.40	1.72	2.00	1.72	2.05	1.59	1.81	1.74	3.14	2.94	2.36	4.91	3.36	2.19	3.00	3.86	2.79	2.43	1.87	0.24	3.10	0.81	0.000215443
**107**	**2.59**	**2.28**	**2.50**	**2.13**	**2.83**	**2.22**	**2.70**	**2.00**	**2.32**	**2.31**	**3.91**	**3.56**	**3.14**	**5.36**	**3.75**	**3.01**	**3.59**	**4.90**	**3.19**	**3.46**	**2.39**	**0.26**	**3.79**	**0.77**	**3.53292E-05**
**108**	**2.35**	**1.87**	**2.00**	**2.01**	**2.15**	**2.05**	**2.67**	**1.81**	**2.09**	**1.90**	**3.38**	**3.10**	**2.76**	**4.35**	**3.64**	**2.39**	**3.01**	**3.83**	**2.99**	**2.78**	**2.09**	**0.25**	**3.22**	**0.58**	**2.38503E-05**
**109**	**2.73**	**1.97**	**2.39**	**2.26**	**2.29**	**2.10**	**2.60**	**2.10**	**2.37**	**2.03**	**3.69**	**3.14**	**2.77**	**3.69**	**3.85**	**2.48**	**3.14**	**4.15**	**3.18**	**2.98**	**2.28**	**0.25**	**3.31**	**0.52**	**2.59149E-05**
**110**	**2.73**	**2.00**	**2.42**	**2.42**	**2.43**	**2.13**	**3.21**	**2.07**	**2.38**	**2.07**	**3.89**	**3.18**	**3.20**	**3.53**	**4.09**	**2.62**	**3.52**	**4.55**	**3.35**	**2.93**	**2.38**	**0.37**	**3.48**	**0.57**	**7.0073E-05**
111	2.84	2.06	2.71	2.63	2.59	2.20	3.42	2.15	2.68	2.11	3.87	3.01	3.25	2.95	4.11	2.65	3.45	4.35	3.36	3.00	2.54	0.42	3.40	0.55	0.00098608
112	3.24	2.38	3.25	3.21	3.12	2.53	4.70	2.50	3.15	2.43	4.44	3.34	3.85	3.27	4.50	3.06	4.12	5.17	3.90	3.45	3.05	0.69	3.91	0.66	0.010914933
113	3.52	2.52	3.47	3.52	3.40	2.71	5.21	2.63	3.49	2.54	4.50	3.43	4.06	3.43	4.62	3.29	4.24	5.02	3.98	3.69	3.30	0.80	4.03	0.58	0.031932173
114	4.13	3.23	4.32	4.22	4.23	3.17	6.47	3.18	4.23	3.10	5.45	4.14	4.46	4.20	5.08	3.73	4.96	5.62	4.58	5.03	4.03	1.01	4.72	0.61	0.077083663
115	3.69	2.44	3.47	3.69	3.30	2.64	4.94	2.55	3.35	2.97	4.55	3.54	3.94	3.56	4.21	3.23	4.10	5.18	3.99	4.64	3.30	0.74	4.09	0.59	0.016060635
116	3.49	2.25	3.04	3.17	2.95	2.46	4.52	2.36	3.21	2.26	3.91	3.07	3.60	2.64	4.21	3.04	3.56	4.23	3.51	3.49	2.97	0.70	3.53	0.51	0.058727213
117	4.23	3.38	4.28	4.34	4.08	3.17	6.52	3.24	4.56	3.12	5.01	3.86	4.38	5.27	5.03	3.77	4.78	5.57	4.45	5.21	4.09	1.01	4.73	0.60	0.102974758
118	3.63	2.62	3.48	4.03	3.49	2.64	6.32	2.69	3.75	2.48	4.21	3.48	3.96	3.51	4.30	3.28	3.81	4.50	3.69	4.95	3.51	1.13	3.97	0.52	0.258953318
119	3.82	2.80	3.47	4.13	3.49	2.71	6.68	2.88	3.87	2.52	4.01	3.46	3.85	3.50	4.47	3.30	3.64	4.34	3.55	4.85	3.64	1.20	3.90	0.51	0.533503224
120	3.73	2.93	3.35	3.69	3.21	2.71	6.55	3.17	3.52	2.51	3.64	3.36	3.49	4.26	4.69	3.12	3.46	4.18	3.28	4.27	3.54	1.13	3.77	0.53	0.553956131

121	4.95	4.14	5.02	4.89	4.85	3.83	7.08	5.64	4.54	3.63	4.83	4.55	4.42	6.36	6.06	5.06	4.09	5.55	4.43	4.85	4.86	0.99	5.02	0.75	0.682958639
122	5.25	4.83	5.80	4.99	4.77	4.46	6.14	7.60	4.47	3.64	4.57	4.78	4.25	7.30	7.30	5.72	4.40	5.31	4.49	4.55	5.20	1.10	5.27	1.16	0.889079728
123	3.72	3.48	4.01	3.73	3.13	3.25	5.30	3.78	3.65	2.53	3.26	4.09	3.48	6.34	6.21	3.72	3.59	4.53	3.58	4.11	3.66	0.71	4.29	1.11	0.14508683
124	3.78	2.98	3.03	3.29	2.77	2.84	4.24	2.81	3.20	2.21	3.04	3.19	2.97	5.54	5.47	3.30	3.07	3.67	3.33	3.82	3.11	0.56	3.74	0.97	0.094657572
125	3.64	2.81	2.81	2.86	2.64	2.79	4.33	2.96	3.16	2.15	2.61	3.05	2.80	6.52	3.93	3.18	2.90	3.28	3.32	3.43	3.02	0.60	3.50	1.12	0.242163109
126	3.06	2.43	2.23	2.42	2.32	2.29	2.85	2.48	3.06	1.91	2.47	2.83	2.67	5.31	3.21	2.50	2.48	2.99	2.99	3.59	2.50	0.37	3.10	0.85	0.056872736
127	3.28	2.41	2.18	2.53	2.50	2.55	2.88	2.52	2.74	1.91	2.65	3.24	3.15	5.60	3.35	2.56	2.62	3.20	3.34	3.07	2.55	0.37	3.28	0.87	0.025891328
128	3.51	2.48	2.40	2.92	2.61	2.85	3.19	2.87	3.02	2.13	3.04	3.33	3.73	5.02	3.75	2.77	3.07	3.42	3.39	3.05	2.80	0.40	3.46	0.63	0.012021177
129	3.64	2.51	2.56	3.12	3.19	3.08	3.25	2.97	3.16	2.42	3.45	4.26	4.35	6.13	3.67	2.84	3.09	3.49	3.97	3.34	2.99	0.38	3.86	0.93	0.014115727
130	2.88	2.09	1.91	2.19	2.30	2.38	2.38	2.23	2.62	1.73	2.60	2.99	3.03	4.88	2.73	2.09	2.30	2.85	3.18	3.33	2.27	0.33	3.00	0.76	0.012672644
131	3.08	2.07	2.32	2.82	2.56	2.66	2.57	2.41	2.73	1.87	3.16	3.02	3.68	3.76	3.30	2.29	2.93	3.42	3.26	2.85	2.51	0.36	3.17	0.43	0.001597132
132	3.01	2.08	2.39	2.53	2.28	2.21	2.37	2.33	2.73	1.90	2.84	2.77	3.10	3.47	3.46	2.19	2.81	3.35	2.70	2.99	2.38	0.32	2.97	0.40	0.001851626
133	2.86	1.96	2.52	2.45	2.23	2.03	2.18	2.10	2.71	1.86	2.87	2.64	3.03	2.67	3.55	2.21	2.74	3.47	2.59	2.77	2.29	0.33	2.85	0.41	0.003128009
134	3.05	2.47	2.91	2.78	2.62	2.35	2.62	2.37	2.96	2.20	3.32	3.04	3.39	3.27	4.25	2.66	3.15	4.15	2.99	3.30	2.63	0.29	3.35	0.49	0.000897892
135	2.92	2.11	2.87	2.80	2.55	2.22	2.85	2.14	2.66	2.08	3.32	2.97	3.33	2.91	4.10	2.84	3.01	4.06	2.86	3.14	2.52	0.35	3.25	0.47	0.000864832
**136**	**3.53**	**2.40**	**3.42**	**3.24**	**3.01**	**2.60**	**2.84**	**2.45**	**2.96**	**2.55**	**3.90**	**4.25**	**4.31**	**4.04**	**4.85**	**3.14**	**3.59**	**4.77**	**3.51**	**4.10**	**2.90**	**0.40**	**4.05**	**0.54**	**4.24197E-05**
137	4.10	2.83	3.55	3.44	3.35	3.07	3.05	2.78	3.30	3.07	5.01	5.72	5.90	5.85	4.50	3.46	4.39	8.07	4.54	3.75	3.25	0.39	5.12	1.34	0.000499001
**138**	**4.12**	**2.89**	**3.88**	**3.61**	**3.53**	**3.30**	**3.16**	**2.83**	**3.48**	**3.27**	**4.31**	**4.61**	**4.83**	**5.68**	**5.13**	**3.50**	**4.21**	**5.86**	**4.05**	**5.01**	**3.41**	**0.40**	**4.72**	**0.74**	**0.000104783**
139	4.25	3.15	4.18	3.90	3.71	3.54	3.15	3.20	3.68	3.70	4.29	4.39	5.03	6.16	6.12	3.76	4.54	5.34	4.12	5.52	3.65	0.40	4.93	0.84	0.000378164
140	3.14	2.27	3.05	2.78	2.71	2.48	2.34	2.29	2.70	2.98	3.53	3.50	4.32	5.53	6.42	2.73	4.16	4.42	3.27	3.56	2.68	0.32	4.14	1.11	0.000807075
141	3.55	2.71	3.55	3.83	3.02	2.87	3.27	2.60	3.14	3.11	3.62	3.93	3.75	5.06	5.53	3.25	3.94	4.41	3.57	4.49	3.17	0.39	4.15	0.71	0.00122979
142	2.56	2.09	2.65	2.38	2.34	2.20	2.23	2.09	2.38	2.01	3.00	2.82	2.64	3.78	3.81	2.58	2.84	3.76	2.64	2.84	2.29	0.21	3.07	0.51	0.000281778
143	2.66	1.95	2.35	2.29	2.13	2.13	2.07	1.86	2.53	1.88	2.63	2.60	2.39	3.48	3.55	2.32	2.60	3.52	2.34	2.62	2.18	0.27	2.81	0.50	0.0029609
144	2.69	1.84	2.20	2.21	1.98	1.85	2.21	1.83	2.31	1.65	2.69	2.37	2.64	2.23	3.22	2.07	2.48	3.27	2.40	2.29	2.08	0.31	2.57	0.40	0.006823367
145	2.31	1.74	1.83	1.99	1.83	1.82	2.09	1.79	2.18	1.51	2.33	2.16	2.35	2.57	2.78	1.87	2.25	2.84	2.17	2.11	1.91	0.24	2.34	0.31	0.002366496
146	2.43	1.64	1.76	2.14	1.74	1.98	2.23	1.88	2.11	1.44	2.54	2.14	2.64	2.90	2.54	1.77	2.34	2.78	2.41	1.94	1.93	0.30	2.40	0.36	0.005597037
147	2.04	1.33	1.54	1.82	1.41	1.42	1.65	1.50	1.96	1.17	2.69	2.25	1.93	2.77	2.03	1.43	1.79	2.25	1.71	1.76	1.58	0.28	2.06	0.43	0.008741227
148	2.45	1.63	1.87	2.28	1.74	1.92	2.14	1.95	2.16	1.57	2.77	2.63	2.53	3.49	2.29	1.84	2.40	2.71	2.66	2.14	1.97	0.28	2.54	0.44	0.00284898
149	2.95	1.93	1.99	2.44	2.15	2.23	2.59	2.25	2.34	1.70	2.86	2.88	3.10	4.15	2.76	2.10	2.55	2.95	3.25	2.67	2.26	0.36	2.93	0.53	0.003791548
150	2.19	1.54	1.73	1.92	1.63	1.82	2.00	1.82	1.91	1.36	2.31	2.22	2.27	3.17	2.14	1.59	1.91	2.16	2.10	2.16	1.79	0.24	2.20	0.40	0.011762689
151	2.06	1.50	1.61	2.13	1.40	1.63	1.77	1.77	2.29	1.38	1.98	1.92	2.02	2.94	1.88	1.53	1.73	1.78	1.84	2.39	1.75	0.31	2.00	0.40	0.14133002
152	2.62	1.64	2.32	2.15	1.75	2.06	1.98	2.27	2.26	1.64	3.28	2.57	2.47	4.61	2.50	1.81	2.46	2.43	2.59	2.25	2.07	0.32	2.70	0.76	0.027152411
153	3.01	1.76	1.85	2.25	2.12	2.24	2.37	2.29	2.12	1.62	4.26	3.18	2.71	4.90	2.51	1.98	2.57	2.84	2.86	2.44	2.16	0.39	3.03	0.89	0.01153616
154	2.67	1.71	1.76	2.16	2.08	3.85	3.16	2.51	1.99	2.31	3.76	3.75	3.30	4.23	2.29	2.02	4.10	3.81	2.65	2.02	2.42	0.66	3.19	0.87	0.038262707
155	2.34	1.77	2.08	2.23	1.83	3.56	2.45	2.63	2.05	1.92	2.45	2.92	2.64	4.30	2.31	1.85	3.54	3.01	2.38	2.19	2.29	0.53	2.76	0.72	0.110562325
156	3.90	2.60	2.71	3.26	2.47	4.60	2.97	2.98	2.98	2.35	4.06	4.11	3.00	6.40	3.36	2.64	5.24	3.73	3.56	3.15	3.08	0.69	3.92	1.13	0.059951885
157	3.49	2.25	2.54	2.87	2.42	4.75	2.99	2.90	2.61	2.35	4.09	4.74	2.87	6.03	2.96	2.36	5.48	4.23	3.38	2.57	2.92	0.74	3.87	1.26	0.053885667
158	2.87	2.48	2.88	3.05	2.83	6.25	3.93	3.27	2.65	2.73	3.73	4.75	3.27	5.05	2.97	2.56	6.40	4.89	3.07	2.49	3.29	1.11	3.92	1.29	0.262638058
159	3.47	2.89	3.53	4.20	2.61	6.98	3.81	3.45	3.26	2.74	3.16	4.72	3.30	6.53	3.52	2.80	7.20	5.11	3.53	3.47	3.70	1.25	4.33	1.51	0.317769529
160	4.43	3.25	3.92	4.17	3.12	7.10	3.62	3.56	3.84	3.10	4.86	6.08	3.39	7.95	3.99	3.21	8.20	5.81	4.35	3.82	4.01	1.17	5.17	1.80	0.106551629
161	4.05	3.24	4.18	4.23	3.15	7.94	4.19	3.91	3.70	3.45	4.74	6.69	3.68	7.22	3.90	3.24	8.93	6.43	4.06	3.39	4.21	1.37	5.23	1.95	0.192227245
162	3.76	3.49	4.61	4.67	3.09	9.36	4.72	4.22	3.85	3.63	3.88	6.52	3.90	6.80	4.04	3.40	9.79	6.92	3.76	3.86	4.54	1.78	5.29	2.11	0.403963259
163	3.73	5.27	4.28	4.60	3.72	8.93	6.23	4.46	5.41	4.17	4.35	6.44	4.18	6.06	3.92	5.71	8.68	6.59	3.38	3.82	5.08	1.56	5.31	1.67	0.751332247
164	2.94	7.31	5.21	3.87	5.66	8.56	5.74	5.12	7.58	4.54	3.84	5.95	4.90	6.38	4.00	7.95	8.19	6.11	3.54	6.09	5.65	1.74	5.69	1.62	0.956933803
165	4.41	9.32	7.23	5.38	8.59	10.89	8.46	6.79	9.83	5.98	4.79	6.94	6.97	7.86	6.10	10.54	9.47	6.62	5.19	8.21	7.69	2.08	7.27	1.80	0.634845463
166	5.12	10.49	8.06	5.32	7.76	9.50	8.90	7.19	11.49	6.75	5.04	8.16	7.77	9.14	7.83	13.21	9.32	7.19	6.30	7.28	8.06	2.09	8.12	2.19	0.946854841
**167**	**5.98**	**11.16**	**9.39**	**6.30**	**8.73**	**9.92**	**11.61**	**8.38**	**13.05**	**8.71**	**0.01**	**0.01**	**0.02**	**0.04**	**0.03**	**0.03**	**0.02**	**0.02**	**0.02**	**0.02**	**9.32**	**2.23**	**0.02**	**0.01**	**1.08735E-10**
